# Unraveling the genomic landscape of piscine myocarditis virus: mutation frequencies, viral diversity and evolutionary dynamics in Atlantic salmon

**DOI:** 10.1093/ve/veae097

**Published:** 2024-11-21

**Authors:** Racheal Amono, Turhan Markussen, Vikash K Singh, Morten Lund, Farah Manji, Sunil K Mor, Øystein Evensen, Aase B Mikalsen

**Affiliations:** Department of Paraclinical Sciences, Norwegian University of Life Sciences, Post box 5003, Ås 1432, Norway; Department of Paraclinical Sciences, Norwegian University of Life Sciences, Post box 5003, Ås 1432, Norway; Department of Veterinary Population Medicine and Veterinary Diagnostic Laboratory, University of Minnesota, 1333 Gortner Avenue, St. Paul, MN 55108, United States; PatoGen AS, Rasmus Rønnebergs Gate 21, Ålesund 6002, Norway; Mowi ASA, Post box 4102, Bergen 5835, Norway; Department of Veterinary Population Medicine and Veterinary Diagnostic Laboratory, University of Minnesota, 1333 Gortner Avenue, St. Paul, MN 55108, United States; Department of Veterinary and Biomedical Sciences and Animal Disease Research & Diagnostic Laboratory, South Dakota State University, Post box 2175 University Station, Brookings, SD 57007, USA; Department of Paraclinical Sciences, Norwegian University of Life Sciences, Post box 5003, Ås 1432, Norway; Department of Paraclinical Sciences, Norwegian University of Life Sciences, Post box 5003, Ås 1432, Norway

**Keywords:** piscine myocarditis virus, diversity, phylogeny, genomic sequencing, defective viral genomes

## Abstract

Over a decade since its discovery, piscine myocarditis virus (PMCV) remains a significant pathogen in Atlantic salmon aquaculture. Despite this significant impact, the genomic landscape, evolutionary dynamics, and virulence factors of PMCV are poorly understood. This study enhances the existing PMCV sequence dataset by adding 34 genome sequences and 202 new ORF3 sequences from clinical cardiomyopathy syndrome (CMS) cases in Norwegian aquaculture. Phylogenetic analyses, also including sequences from the Faroe Islands and Ireland revealed that PMCV sequences are highly conserved with distinct clustering by country of origin. Still, single CMS outbreaks display multiple PMCV variants, and although some clustering was seen by case origin, occasional grouping of sequences from different cases was also apparent. Temporal data from selected cases indicated increased sequence diversity in the population. We hypothesize that multiple bottlenecks and changing infection dynamics in the host population, with transfer to naïve individuals over time, represent a continuous selection pressure on the virus populations. No clear relation was found between PMCV variants and the severity of heart pathology. However, specific non-synonymous and synonymous mutations that might impact protein function and gene expression efficiency were identified. An additional factor that may impact PMCV replication is the presence of defective viral genomes, a novel finding for viruses of the order *Ghabrivirales*. This study provides new insights into PMCV genomic characteristics and evolutionary dynamics, highlighting the complex interplay of genetic diversity, virulence markers, and host-pathogen interactions, underscoring the epidemiological complexity of the virus.

Keywords: piscine myocarditis virus; evolutionary dynamics; diversity; phylogeny; genomic sequencing; defective viral genomes

## Introduction

Piscine myocarditis virus (PMCV) infects Atlantic salmon (*Salmo salar* L.) in aquaculture, resulting in cardiomyopathy syndrome (CMS). The infection and disease have traditionally been seen in adult fish in the second year at sea, but occasionally, it has also been shown in young post-smolts a few months after sea transfer. The disease has been found in Norwegian Atlantic salmon since 1988 (Amin and Trasti 1988) and subsequently found in the Faroe Islands (Poppe and Seierstad 2003), Scotland (Rodger and Turnbull 2000), and Ireland (Rodger et al. 2014). A disease indicative of CMS has also been described in Canada (Brocklebank and Raverty 2002). The most typical clinical signs are an enlarged atrium and sinus venosus, and a ruptured atrium wall, resulting in cardiac tamponade with blood filling the pericardial cavity. Histologically, lesions are first observed in the atrium and subsequently in the ventricle, and include sub-endocardial inflammation, myocarditis, and in severe cases, degeneration and necrosis of spongious myocardium (Amin and Trasti 1988, Ferguson et al. 1990, Fritsvold et al. 2009).

PMCV was first characterized in 2011 (Haugland et al. 2011). It is a double-stranded RNA virus with a genome length of 6.7 kb, including three open reading frames (ORFs 1–3). The first homology investigations showed that ORF2 has similarity to the ORF2 encoding an RNA-dependent RNA polymerase (RdRp) of the *Totiviridae* family, order *Ghabriviriales*, infecting single-celled protozoa. The closest relation was seen to Giardiaviruses (Haugland et al. 2011). However, homology has also been observed for both ORF1 (encoding capsid) and ORF2, to viruses of an increasing group first described as “toti-like viruses” found in more advanced hosts, like various terrestrial and aquatic arthropods (Wu et al. 2010, Zhai et al. 2010, Isawa et al. 2011, Shi et al. 2016, Prasad et al. 2017, Bačnik et al. 2021), bats (Kohl et al. 2021, Couto et al. 2024), and in addition to Atlantic salmon hosting PMCV, also other fish species (Mor and Phelps 2016a, b, Sandlund et al. 2021, Louboutin et al. 2023). The order *Ghabriviriales* was recently reorganized and four piscine viruses characterized by additional ORF(s) in 3ʹ end of genome, including PMCV, were assigned to a new virus family named *Pistolviridae* (Simmonds *et al.* 2024). A −1 ribosomal frameshift site is present at the far 3ʹ end of ORF1, a general characteristic of the *Ghabriviriales* order (Simmonds *et al.* 2024). For the totivirus L-A virus, the frameshift is described to happen at an ∼1.8% frequency. In these events, capsid is translated beyond ORF1 into the overlapping ORF2, resulting in a few copies of a capsid–RdRp fusion protein per virus particle (Dinman et al. 1991). Similar has also been shown for viruses of *Artiviridae* infecting arthropods (Okamoto et al. 2016). Due to the presence of the −1 ribosomal frameshift site in PMCV ORF1, the translation of capsid–RdRp proteins is also expected to occur for PMCV, but this has not been shown experimentally. A putative ORF2 start codon is also present 83 nucleotides downstream of ORF1 (Haugland et al. 2011, Sandlund et al. 2021). PMCV ORF3 encodes a protein with no shared sequence homology to proteins of other viruses or other known proteins, except a small N-terminal domain showing homology to chemokines (Haugland et al. 2011, Sandlund et al. 2021). The ORF3-encoded protein has a predicted size of 33.4 kDa and is currently named p33.

Previous sequence studies of PMCV in Atlantic salmon have been performed on concatenated ORF1 and ORF3 sequences achieved from Sanger sequencing. These have shown that Norwegian strains are highly similar, and it has been suggested that they all belong to a single genogroup (Wiik-Nielsen et al. 2012). Sequence variation between individuals of single outbreaks was observed, and although the phylogenetic analyses showed some spatially associated clustering, sequences from individual fish from the same outbreak could also disperse throughout the phylogenetic tree, complicating the epidemiological analyses. Similar studies of Irish strains confirm this (Tighe et al. 2019). A higher sequence variability in ORF3, compared to ORF1, was indicated for PMCV from Norway and Ireland in these studies (Wiik-Nielsen et al. 2012, Tighe et al. 2019).

Through the decade since the initial discovery of the virus, only one fullgenome sequence has been available, i.e. the Norwegian AL V-708 isolate (Haugland et al. 2011), and there has also been a lack of separately published sequences for ORF2 and the untranslated regions (UTRs).

Increasing the availability of sequences represents a first and essential step in evaluating any evolutionary paths of PMCV better and attempting to understand the potential linkages between different PMCV variants, infection, and disease. In this study, we present 34 new complete or near-complete PMCV full-genome sequences from Norway achieved through next-generation sequencing of RNA. These new genome sequences originate from eight seawater salmon aquaculture sites in five Norwegian counties sampled in 2011, 2017, and 2018. We also include 11 near-complete genome sequences from the Faroe Islands, including one from wild salmon. All fish were sampled from cases confirmed to have clinical CMS with a few exceptions. Additional PMCV ORF3 sequences were obtained from individuals from the same cases and from a time-course sampling from four other cases with variable degrees of clinical CMS from 2019 to 2020.

Phylogenetic and detailed sequence analyses were performed on the full-genome sequences and for individual ORFs from all cases, including all currently available PMCV sequences in GenBank. Several parameters related to sequence diversity are also described and compared for the various ORFs and UTRs, and for ORF3 in the time-course studies. In addition, we have evaluated the presence of specific mutations or mutation motifs, combined with data on viral load and heart lesion severity per individual and/or mortality at population level, to reveal any factors essential to the virulence of PMCV.

## Materials and methods

### Field case descriptions—multiple cases (Cases A– H)

**Figure 1.**
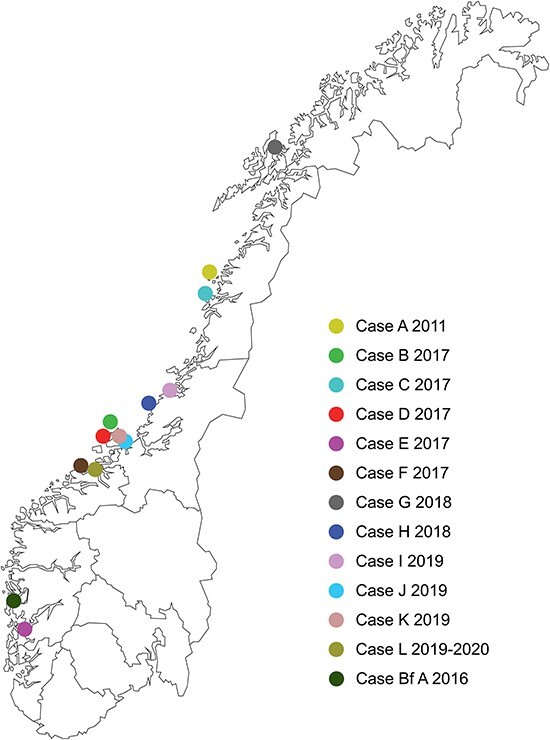
Map of Norway showing geographic location of the seawater Atlantic salmon aquaculture sites of cases included in the study. The cases (Cases A–L and Bf A) are color-coded by case origin and with year of sampling, as indicated.

Samples were collected from Atlantic salmon (*Salmo salar* L.) in eight Norwegian seawater aquaculture sites (Cases A–H). The earliest sample set was from a seawater site in Nordland County, sampled in 2011 (Case A), available from an internal field sample depository. The remaining seven were collected between 2017 and 2018 (Cases B–H) (Table 1, Fig. 1). For Case D, samples were obtained at three different time points (Cases D1–D3). In addition, samples were collected from a Norwegian broodfish population at a breeding facility in 2016 (Case Bf A). A selection of the field sites clusters in the county of Trøndelag, but sites from almost all salmon-producing counties were included (Fig. 1).

**Table 1.** Norwegian CMS cases in the study with sampling time point, geographic location of aquaculture sites, disease information, and fish condition upon sampling.

Case	Sampling (month/year)	Geographic location (County)	CMS status in the population	Additional disease information	Fish condition of sampled individuals	Number of individuals with resulting full genome^e^
Field outbreak - production sites						
A	04/2011	Nordland	Clinical outbreak		n.a.	4
B	06/2017	Trøndelag	Clinical outbreak	Clinical PD	Dead	6
C	06/2017	Nordland	Clinical outbreak	Clinical HSMI	Moribund	2
D1^a^	07/2017	Trøndelag	^b^		n.a.	-
E	08/2017	Vestland	Clinical outbreak		Moribund	2
D2^a^	08/2017	Trøndelag	Possible^b^	^b^	Moribund	3
F	09/2017	Møre og Romsdal	Clinical outbreak		n.a.	1
D3^a^	12/2017	Trøndelag	Possible^b^	Possible PD/HSMI^b^	Moribund	3
G	01/2018	Troms og Finnmark	Clinical outbreak		Moribund	1
H	12/2018	Trøndelag	Clinical outbreak		Moribund (F2–4), Dead (F1, 5–12)	12
I	08–11/2019^c^	Trøndelag	No clinical outbreak		Moribund/dead^d^	-
J	04–10/2019^c^	Trøndelag	Clinical outbreak		Moribund/dead^d^	-
K	03–09/2019^c^	Trøndelag	Clinical outbreak		Moribund/dead^d^	-
L	09–12/2019 + 01/2020^c^	Møre og Romsdal	Clinical outbreak		Moribund/dead^d^	-
Field outbreak - brood fish site						
Bf A	01/2016	Vestland	Clinical outbreak		Unknown	-
Challenge trials						
Ex A	07/2007		Clinical outbreak	No disease	Healthy	-
Ex B	05–07/2018		Clinical outbreak	No disease	Healthy	10 per sampling

a For Case D, samples were obtained at three different time points (Cases D1–D3).

b For Case D1, only a low number of PMCV-positive samples, also of low viral load as determined by real-time PCR, were observed. At D2, histological lesions were described as a possible mix of CMS and HSMI. Piscine orthoreovirus was detected at 100% prevalence with viral RNA levels consistent with HSMI. In addition, there was a 10% prevalence of salmonid alphavirus at low viral RNA levels, indicating early stages of pancreas disease (PD). At D3, histological changes were described as CMS, possibly mixed with HSMI and/or PD.

c Monthly samplings.

d Fish were sampled as either moribund or dead, but this information was unavailable at the individual level.

e Samples from additional individuals were also subjected to Sanger sequencing of PCR amplicons of ORF3. For D1, only partial ORF3 sequences were obtained from two fish. From Cases I–L and Ex A, only ORF3 sequences were obtained, by Sanger sequencing of PCR amplicons. In Ex B, only partial genome sequences were obtained from RNA sequencing.

n.a.—information not available.

During sampling, authorized fish health personnel evaluated all cases for diseases. CMS diagnosis was confirmed for most cases. Case D had no disease diagnosis in the first sampling (D1) but CMS was suspected to be mixed with heart and skeletal muscle inflammation (HSMI) and/or pancreas disease (PD) in the two later samplings (D2 and D3). Fish individuals were sampled as either moribund or dead. Details on the sampling time point, geographic location of the aquaculture sites, disease information, and fish condition at sampling may be found in Table 1. In all cases, all fish were sampled from a single cage.

### Field case descriptions—time course studies (Cases I–L)

Samples from time course studies of the presence of PMCV in Atlantic salmon (*Salmon salar* L.) at four Norwegian seawater aquaculture sites performed externally were made available (Cases I–L) (Table 1, Fig. 1). Moribund/dead fish individuals were screened for PMCV monthly from sea transfer to slaughter using a PCR diagnostic service. PMCV was detected approximately one year after sea transfer at all four sites. All study populations included were evaluated for diseases by authorized fish health personnel, and a CMS diagnosis was set for Cases J–K, including CMS-specific mortality. In each case, the study population included individuals originating in single smolt groups, sampled from a set of cages at the site. Fish were randomly sampled as moribund or dead fish from the various cages representing the study population.

### Experimental challenges

Samples from two experimental trials of Atlantic salmon (*Salmo salar* L.) challenged with PMCV have been included (Table 1). Samples from Experimental challenge A (Ex A) originate from a previous non-published study in collaboration with external partners. The challenge material was a parallel aliquot sample of the cell culture supernatant used in a previously published challenge (Haugland et al. 2011). Atlantic salmon parr (mean weight 30 g) were held in fresh water at 8°C and challenged by intramuscular injection, as previously described (Haugland et al. 2011). One aliquot of the challenge material and heart tissue sampled from one individual at 16 weeks post challenge (wpc) were included in the present study.

Tissue samples from Experimental challenge B (Ex B) originate from a second previous non-published experimental challenge performed by internal collaboration with external partners in a non-related project. Challenge material was prepared from heart tissue samples from individuals F12, F16, and F20 in Case G; all indicated a high load of PMCV and no detectable presence of the ubiquitous piscine orthoreovirus causing HSMI from real-time PCR analyses. The three tissue pieces were homogenized in Leibovitz’s L-15 medium (Gibco) w/ 100 µg/ml gentamycin at a 1:10-dilution (weight/volume), centrifuged at 2500 rpm for 10 min at 4°C to remove cell debris, and the supernatant sterile filtered through a 0.45 μm filter. A total of 60 unvaccinated Atlantic salmon (mean weight 75 g) were used in the experiment. The fish had been smoltified (seawater adapted) according to standard procedures of the aquarium facilities (Havbruksstasjonen i Tromsø AS, Tromsø, Norway) and were kept in 25‰ salt water. The fish were fed according to standard procedures. The water temperature was maintained at 10°C. The fish challenge was done by intraperitoneal injection of 0.1 ml of the filtered heart homogenate per fish under anesthesia [Tricaine Pharmaq® (tricaine mesylate, “MS222”)]. Challenge material (tissue homogenate supernatant) and heart tissue samples on RNAlater from 10 individuals at 6, 8, and 10 wpc were available for RNA extraction and sequencing analyses.

### Sampling of fish and tissue and preservation of samples

In the field, live fish were sampled as moribund fish from the surface and immediately killed by a blow to the head followed by decapitation. Deceased fish were selected from the accumulated dead fish pool at the bottom of the cage. Some deceased fish were degraded to varying degrees due to the varying post-mortem times. From experimental challenges, the individuals were sampled from the tanks using a dip net and anesthetized in Tricaine Pharmaq® (tricaine mesylate, “MS222”) baths. Sedated fish were killed by decapitation before sampling.

For molecular analyses targeting PMCV RNA, an ∼3 mm × 3 mm × 3 mm piece of the ventricle vertex was fixed in RNAlater (Ambion) at 22°C for ∼24 h before storage at −20°C. For Cases A, B, D–F, H, and Ex A and B, a longitudinal incision of the heart was performed, and one part, including atrial tissue, was fixed in 10% neutral phosphate-buffered formalin and stored at room temperature for later preparation for histological analyses.

### Histology

Formalin-fixed tissues were processed for histological examination following standard procedures and stained with hematoxylin and eosin (Bancroft and Stephens 1990). Histopathological examination of the tissues was performed “blind” with no knowledge of infection or disease status. The cardiac atrium and compact and spongy layers of the ventricle were evaluated separately, and CMS characteristic lesions were scored according to previous published (Fritsvold et al. 2009).

### RNA extraction

Several extraction procedures have been used to extract RNA (or total nucleic acids) from the tissue samples. Extraction for real-time PCR testing and PCR amplification for Sanger sequencing was performed using either a manual procedure including RNeasy® Fibrous Tissue Mini Kit (Qiagen) (Cases A, B, E, H, and ExA) or automized procedure including Reliaprep simplyRNA HT system (Promega) on Biomek 4000 (Beckman Coulter) (Cases C, D, F, and G). The initial homogenization of each tissue was done in the kit lysis buffers using a FastPrep-24 homogenizer (MPBiomedicals) for 2 min at 20 Hz. The isolated RNA was quantified using the NanoDrop ND-1000 spectrophotometer (NanoDrop technologies) or Agilent 2100 Bioanalyzer (Agilent technologies).

Nucleic acids from samples related to Cases Bf A and I–L were extracted from the tissue samples using commercial fish diagnostic services (Pharmaq Analytiq AS, Bergen, Norway or PatoGen AS, Ålesund, Norway) and submitted to the laboratory.

RNA subjected to next-generation sequencing (NGS), RNA sequencing (RNA seq), was extracted from tissue samples parallel to those used for real-time PCR or Sanger sequencing (Cases A–H and Ex B). Tissue samples were homogenized using a stomacher and centrifuged at 3200 *g* for 15 min to get clear supernatants for RNA extraction. RNA was then isolated using the QIAamp Viral RNA Kit (Qiagen) following the manufacturer’s instructions, excluding the carrier RNA addition option.

RNA from cell culture supernatant used as challenge material in Challenge Ex A was extracted using QIAamp Viral RNA kit (Qiagen) after the manufacturer’s instructions, excluding the carrier RNA addition option. A complete overview of the experimental handling of individual samples is provided in Supplementary Table S1.

### Real-time PCR

For Cases C, D, F, and G, a one-step reverse transcriptase real-time PCR was run on the RNA to verify the presence and levels of PMCV-specific RNA in the samples, using QuantiFast SYBR Green RT-PCR Kit (Qiagen) together with 2 µl RNA template and primers CMS-qPCR-F4 and CMS-qPCR-R4 (all primer sequences are given in Supplementary Table S2).

Alternatively (Cases A, B, E, and H), a two-step procedure was performed using SuperScript^TM^ III Platinum®Two-Step qRT-PCR Kit with SYBR® Green (Invitrogen) according to the manufacturer’s instructions and with primers and conditions as previously described (Haugland et al. 2011; Supplementary Table S2) using 1 µg RNA in a 20 µl reaction in the first reverse transcriptase step and subsequently, 2 µl of 1:2 diluted cDNA was used as template in the PCR step.

All real-time PCRs were run using a Light Cycler ® 96 (Roche) after the manufacturer’s procedures, including combined annealing and extension at 60°C for 40 cycles.

All real-time PCR related to the time-course PMCV field studies of Cases I–L were performed using the commercial fish diagnostic services offered by PatoGen AS, Ålesund, Norway, as earlier described (Mikalsen et al. 2020). A complete overview of real-time PCR used for individual samples is provided in Supplementary Table S1.

### RNA sequencing

The extracted RNA was submitted to the University of Minnesota Genomic Centre (UMGC) for quality assurance, library preparation, and RNA seq. Libraries were made using the SMARTer® Stranded Total RNA-Seq Kit v2, following the instructions from the manufacturer (Clontech/Takara Bio). The final libraries were normalized using standard library quantification and quality control procedures recommended by Illumina, San Diego, CA, USA. Sequencing from field Cases A–G was performed on a NovaSeq 6000 platform (2x150 PE) and field case H and sample from experimental challenge B (Ex B) on a NextSeq 2000 platform (2x75 PE).

### Sanger sequencing of PMCV ORF3 plasmid clones

A total of 11 µl of RNA extracted from the supernatant of the challenge material in the PMCV challenge experiment Ex A and 1 µg of RNA extracted from the heart tissue of fish from 16 wpc were subjected to cDNA synthesis using SuperScript®III reverse transcriptase kit (Invitrogen), including random hexamer primers in a total volume of 20 μl. The procedures were all performed after manufacturer’s instructions, except for the initial denaturation of RNA prior to cDNA synthesis, which was performed at 95°C for 2.5 min to increase the denaturation of strong secondary structures. The resulting cDNA was used to amplify PMCV ORF3 using a DyNAzyme EXT DNA polymerase kit (Thermo Scientific) and available primers (Supplementary Table S2). Resulting PCR products were separated through gel electrophoresis and purified using QIAquick Gel Extraction kit (Qiagen) and subsequently ligated into the pCR 2.1 vector using a Topo TA cloning kit (Invitrogen) and transformed into competent OneShot TOP10 bacterial cells (Invitrogen) and cultured overnight at 37°C on LB-agar plates including 50 µg/ml kanamycin. Ten bacterial clones originating in either ORF3 from challenge material or heart tissue at 16 wpc were grown in LB-medium containing 50 µg/ml kanamycin. Plasmids were purified using a QIAprep mini spin kit (Qiagen), and the ORF3 insert was sequenced using primers binding to the M13 site in the plasmid backbone using a commercial service (GATC).

### Sanger sequencing of PMCV ORF3 PCR amplicons

For samples originating in Cases A–F, 1 μg of total RNA was subjected to cDNA synthesis using a Transcriptor first-strand cDNA kit (Roche) with a mixture of oligo(dT) and random hexamer primers in a total volume of 20 μl. Complete ORF3s were sequenced by amplification of two partially overlapping ORF3 gene fragments using the AccuPrime Taq DNA Polymerase System (Invitrogen) with a standard PCR protocol with the primer pairs PMCV-ORF3-F1/-R1 and -F2/-R2 (Supplementary Table S2). A shorter product was made by amplification using PMCV-HMR-F1 and R1 primers (Supplementary Table S2) for samples where full-length sequences were difficult to obtain. The PCR products were gel purified using a QIAquick Gel Extraction kit (Qiagen) and sent to a commercial service for Sanger sequencing using the primers used for amplification (Eurofins GATC). For samples originating in Cases I–L, 11 µl total nucleic acids were subjected to cDNA synthesis and subsequent PCR amplification procedure using SuperScript®IV reverse transcriptase kit (Invitrogen), including random hexamer primers for cDNA synthesis and Platinum™ SuperFi II DNA Polymerase (Invitrogen) for PCR. The procedures were all performed after manufacturer’s instructions, except for the initial denaturation of RNA prior to cDNA synthesis, which was performed at 95°C for 2.5 min to increase the denaturation of strong secondary structures.

### Sanger sequencing for identification of PMCV RNA deletion variants

PMCV-specific RNA, including deletions, was discovered by coincidence through the above-described Sanger sequencing of PMCV ORF3 amplicons. In addition, PCR amplicons appearing shorter than the expected size after gel electrophoresis were selected from these PCRs and analyzed for the presence of deletions by ligation of amplicon into the pCR 2.1 vector using TA-cloning and subsequent sequencing, all as described earlier. PCRs were also designed to amplify larger parts of the genome (see primer information and specificity and amplicon size in Supplementary Table S2) and analyzed similarly for the presence of deletions in products appearing shorter than expected.

### Bioinformatics—analyses of raw data from RNA-seq and Sanger sequencing

The quality of the RNA seq data was evaluated using FastQC and/or MultiQC. Raw reads were processed to remove adapters and low-complexity sequences using Trimmomatic (v 0.39) with a minimum quality score of 30. Trimmed and cleaned reads were further aligned against the host genome using Bowtie2 (v 2.3.5). Aligned host reads were filtered, and remaining unmapped reads were used for assembly with SPAdes (v3.13.0) with k-mer values of 21, 31, 41, 51, 61, and 71, and the option-careful with a minimum coverage of 5 reads per contig. Then, contigs were compared to the RefSeq viral and non-redundant protein reference databases using Diamond BLASTx at an e-value of 1e^−5^ for significant hits. Genomes were assembled through reference-based assembly using Bowtie2 (v 2.3.5) and the PMCV reference isolate AL-V708 genome sequence (GenBank accession no. HQ339954).

To determine sequence variant frequencies, we used Lofreq (Wilm et al. 2012) after mapping the reads on the consensus sequences obtained from whole-genome sequencing with Bowtie2 (v 2.3.5). To avoid false positives, only single-nucleotide intra-host variation frequencies (iSNVs) of 2% or higher frequencies were included as measures of intra-host virus diversity (Fusaro et al. 2016; McCrone and Lauring 2016, Zhao and Illingworth 2019). A Lofreq default setting of 10 for the minimum read depth was used.

All Sanger sequencing analyses of chromatograms, sequence assemblies, and general nucleotide and amino acid sequence analyses were performed using CLC Main workbench 22.0.2 (Qiagen) and Vector NTI 11.0 (Invitrogen).

### Bioinformatics—general analyses

The number of sequence variants and variant diversity of full genomes and in the separate genomic segments of ORFs and UTRs were calculated using Dnasp v6 software (Rozas et al. 2017) and given from the software’s resulting values of haplotypes (h) and haplotype (gene) diversity (Hd), respectively. The software was additionally used to calculate nucleotide diversity (π), Tajima’s D, and dN/dS values. The analyses were performed utilizing the option of which positions with missing data (gaps) were excluded. A parallel control analysis was included for analyses of π using the pairwise deletion option, in which positions with missing data were considered and analyzed at individual positions (column by column), which produced similar results. For all analyses, the sequence of ORF2 is defined as in PMCV reference isolate AL-V708 genome sequence (GenBank accession no. HQ339954), i.e. from the putative start codon 83 nucleotides downstream of stop codon of ORF1. The 83 nucleotides linking ORF1 and ORF2 in the possible event of a frameshift causing capsid–RdRp fusion protein, is here named link^1−2^ and analyzed separately.

The secondary structure of PMCV ORF3 RNA with upstream and downstream UTRs (nt 5295–6688 of PMCV AL V-708 reference genome) was predicted using Mfold v2.3 available at http://www.unafold.org/mfold/applications/rna-folding-form-v2.php (Zuker 2003). Default settings were used in all the predictions, except for temperature, which was set to 15°C. The predicted structure producing the lowest Gibbs free energy change (ΔG) value was selected.

### Phylogenetic analyses

Multiple sequence alignments were performed using AlignX implemented in the Vector NTI 11.0 software package (Invitrogen), CLC Main workbench 22.0.2 (Qiagen), and MEGA X version 10.1.7 (Kumar et al. 2018). Separate phylogenetic trees of full genome sequences and ORFs 1-3 were generated with the MEGA X software using maximum likelihood (ML) and the TN93 + G model of nucleotide substitution, the most appropriate model suggested by the software for all four trees. Bootstrap values were calculated from 1000 replicates and values above 70 were considered significant (Hillis and Bull 1993, Hungnes et al. 2000). The ML tree for PMCV genomes from Norway and the Faroe Islands was generated from 46 mostly complete genome sequences (6688 nucleotides). However, some sequences with incomplete 5ʹ and 3ʹ ends and/or minor internal gaps were also included, the shortest sequence being 6325 nucleotides in length (D2 Tr2017-F15).

The ML tree of PMCV ORF3 was generated from 251 sequences from Norway, including 159 new sequences obtained from Cases A–L and Bf A and sequences available from GenBank from Norway, the Faroe Islands and Ireland. The tree was constructed with complete ORF3 coding sequences (909 nucleotides). A few sequences from Norway were incomplete in 5ʹ- and 3ʹ-end gaps. The shortest sequence used was 825 nucleotides in length (B Tr2017-F6). All ORF3 sequences from Ireland were partial with incomplete 5ʹ and 3ʹ ends, ranging from 600 to 681 nucleotides in length. Two ORF3 sequences from Norwegian wild Atlantic salmon available from GenBank were also partial, both with lengths of 699 nucleotides.

The PMCV ORF1 ML tree was generated from complete (2583 nucleotides) or nearly complete sequences from Norway and the Faroe Islands. The sequences include the complete ORF1s extracted from the full-genome sequencing of Norwegian and Faroes samples from the present study and Norwegian sequences from GenBank. Some sequences had minor internal gaps; the shortest sequence was 2255 nucleotides long (D2 Tr2017-F15).

Similarly, the PMCV ORF2 ML tree was made from complete (2181 nucleotides) or nearly complete sequences, extracted from the full-genome sequences of Norwegian and Faroese samples available from this study, with the addition of the only ORF2 sequence available in GenBank from the reference sequence from PMCV isolate AL V-708. Five of these sequences had minor internal gaps, the shortest being 2060 nucleotides in length (E Ve2017-F2). As for other analyses, the sequence of ORF2 is defined as in PMCV reference isolate AL-V708 genome sequence (GenBank accession no. HQ339954), i.e. from the putative start codon 83 nucleotides downstream of stop codon of ORF1.

For display purposes, all four trees were rooted against the wild salmon sequence from the Faroe Islands due to its high divergence towards the other sequences. Accession numbers of all PMCV sequences obtained in the present study and those retrieved from GenBank used for phylogeny with more detailed information on origin are listed in Supplementary Table S3.

### Statistical analysis

Statistical significance of Tajima’s D measures was given from the DnaSP v6 software used. Statistical significance of nucleotide and sequence diversity differences between time points of Cases I–L were analyzed using an ordinary one-way ANOVA test using GraphPad Prism version 10.0.0, including a post-hoc Tukey’s test. We also tested for linear trends between time points, using the first time point of virus detection as reference. Bartlett’s test is included in the GraphPad analysis, testing for homogeneity of variances.

### Ethics statement

All fish used in the study were handled and sacrificed in accordance with the Norwegian Animal Welfare Act, and all efforts were made by skilled personnel to minimize suffering. The field tissue samples of Cases A–H were sampled for this study. To avoid the unnecessary sacrifice of fish individuals, the sampling from some of these sites was performed in collaboration with the external project “Epidemiological study of CMS: Risk factors, Disease development, and Transmission,” funded by the Norwegian Seafood Research Fund, project number 901118, (Svendsen et al. 2019). Samples from Cases I to L were also made available in the internal depositories of coauthors, and samples from Ex B were made available in collaboration with an external partner. All field samples originate from private aquaculture sites. The fish/samples are obtained by donation with permission from the owners to use them. All fish were stunned by a blow to their head and killed by exsanguination. The tissue samples from Ex A are residual samples intended initially for research on CMS in a previous project (by the author Aase B. Mikalsen). The challenge and feed experiment (Ex B) was approved by Forsøksdyrutvalget [The Norwegian Animal Research Authority (NARA)]. The fish were anesthetized with MS222 (tricaine) before vaccination or injection challenge and similarly anesthetized at sampling and killed by a blow to their head before dissection.

## Results

### The PMCV genome is highly conserved, with most mutations appearing at low frequency

In the first part of this study, RNA sequencing (RNA seq) was performed on heart tissue samples of individual Atlantic salmon originating from eight CMS-related disease outbreaks (Cases A–H) from five counties in Norway, sampled in 2011, 2017, and 2018 (Table 1, Fig. 1). Thirty-four complete or near-complete PMCV consensus genomes were obtained from the eight cases, 1–12 individuals/case (see detailed info in Supplementary Table S3).

**Figure 2.**
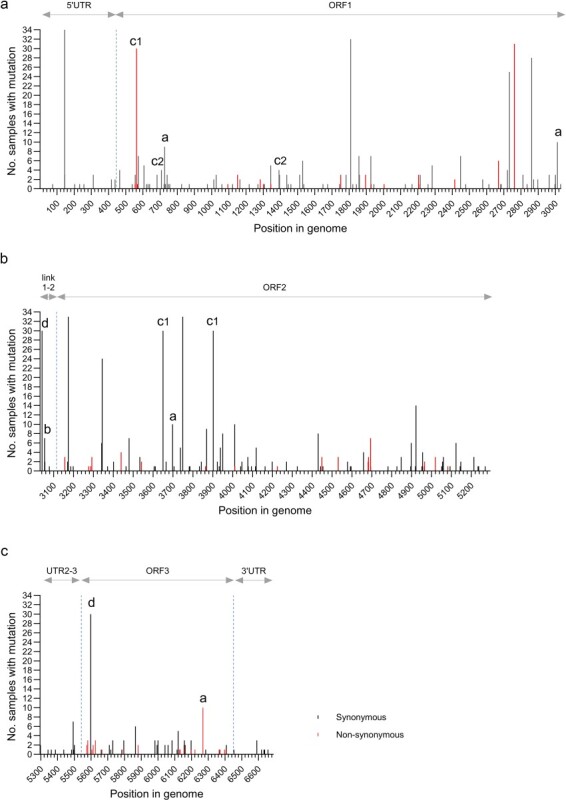
Overview of the positions in the 34 PMCV genomes with diverging nucleotides identified by comparison to PMCV AL V-708 reference genome. Bar height represents the number of genomes with divergent nucleotides in each specific position. Red-colored bars indicate positions resulting in a non-synonymous change, leading to amino acid divergence. A few notable examples of single diverging nucleotides or combinations are marked by a–d. (a) G_6268_C in ORF3, resulting in E_243_Q in p33, is highly prevalent in the dataset and is consistently found concurrent with the synonymous C_726_T and A_3012_G in ORF1 and C_3698_T in ORF2 in the full-length genome dataset. (b) If the putative −1 ribosomal frameshift site at the 3ʹ end of ORF1 is translationally functional, the link^1−2^ is translated as a linker between the capsid and RdRp (Haugland et al. 2011, Sandlund et al. 2021), and this A_3054_G represents a non-synonymous mutation. (c) C_563_T in ORF1, resulting in an A_40_V substitution in the capsid, and the synonymous mutations G_3650_C and G_3902_A in ORF2 (all marked by C1) are examples of the 13 most prevalent mutations. However, a few variants concurrently lack these three mutations. Genomes lacking the C1 marked mutations include a unique combination of the synonymous A_708_G and A_1392_G in ORF1 (both marked by C2). (d) The synonymous mutations A_3041_G and A_5598_G are other examples of high-prevalence mutations. Still, four full-length genomes originating from three cases did not include these two mutations concurrently.

Multiple sequence alignments revealed differing nucleotides in 244 (3.6%) of the 6688 positions in the genome using the earliest available genomic sequence of PMCV AL V-708 from 2007 as the reference (Fig. 2).

Of these variable nucleotide positions, 119 were found only once, i.e. in the consensus genome sequence from a single fish. The remaining 125 positions were seen in multiple individuals, and 79 were found in genome sequences from more than one case. Of these more prevalent 79 mutations, 13 were highly prevalent and present in the majority of the full-genome sequences obtained and found in all cases where PMCV was sequenced from more than two individuals (Fig. 2). Among the 13 highly prevalent mutations, the C_144_T mutation (in the 5ʹ UTR) was present in all new genome sequences. The remaining 12 were found in 24–33 of the new genomes. Noteworthy, all these 13 mutations were also observed in the earliest samples included in this study, originating from 2011 (Case A). Only two highly prevalent mutations are non-synonymous: C_563_T, which results in an A_40_V substitution, and A_2761_G, which results in a T_773_A substitution. Both amino acid substitutions are found in the capsid protein encoded by ORF1. An overview of positions with mutations and their prevalence among individuals is given in Fig. 2, with a selection of notable mutations or mutational patterns highlighted. The consistent occurrence of these mutations within and between individuals across separate cases may suggest they are being selected due to a fitness advantage for the virus and transferred between individuals/cases or are common recurring spontaneous mutations within each case.

The PMCV genome consensus sequence displaying the highest pairwise nucleotide sequence identity to the PMCV AL V-708 reference genome sequence from 2007 (99.8% identity, 15 divergent nucleotides) was identified from a fish from 2017 (Case E/F2). This illustrates that highly similar PMCV genome consensus sequences can be found from outbreaks separated by a decade. The pairwise comparison also revealed that the most divergent variants (Case C/F5 vs. Case F/F8) in the study share 99.2% nucleotides, i.e. only 57 divergent nucleotides were found over the 6688-nt genome (Supplementary Fig. S1).

### Diversity and selection measures vary between individual PMCV coding regions and UTRs

We first attempted to decipher viral genetic diversity and selection pressure using a set of measures to capture different aspects of all available Norwegian sequences of the full genome, the three ORFs and short 83 nucleotides putative ORF1–ORF2 fusion link (link^1−2^), and the UTRs individually (Table 2). The percentages of variable positions of the total number analyzed were lowest in 5ʹ and 3ʹ UTR (2.0 and 2.5%, respectively) and slightly higher in the three ORFs and internal UTR^2−3^. The relatively short link^1−2^ was found to have the highest percentage of variable positions (4.8%). These differences between coding regions and the end UTRs are also reflected in the measures of sequence variants and sequence diversity. Nucleotide diversities (π) were generally low. No significant differences were found between the three ORFs (0.0050–0.0053). The 5ʹ and 3ʹ end UTRs showed the lowest nucleotide diversities, whereas the link^1−2^ showed the highest diversity (0.0090). Tajima’s Ds were negative for the full-length genome and all the ORFs and UTRs (Table 2), although only the values for the 5ʹUTR and ORF3 were given as statistically significant by the software. This suggests the action of purifying selection. Similarly, the low dN/dS ratio obtained for all three ORFs indicates that most non-synonymous mutations are eliminated by purifying selection (Table 2).

**Table 2.** Diversity and selection measures for the PMCV full genome, individual ORFs, and UTRs of the Norwegian genomes.

	Complete genome	5ʹUTR	ORF1/Capsid	link^1−2^	ORF2/RdRp	UTR^2−3^	ORF3/p33	3ʹUTR
Genomic position	1–6688	1–444	445–3030	3031–3113	3114–5294	5295–5541	5542–6450	6451–6688
Sequence length (nts)	6688	444	2586	83	2181	247	909	238
Number of variable nts^a^	244	9	87	4	88	9	41	6
Variable nts of total (%)	3.6	2.0	3.4	4.8	4.0	3.6	4.5	2.5
Sequence variants^b^	32	7	28	6	26	10	25	6
Sequence diversity^b^^,^^c^	0.993	0.363	0.983	0.556	0.968	0.629	0.965	0.360
SD of Seq. div.	0.009	0.104	0.013	0.094	0.020	0.089	0.020	0.101
Nucleotide diversity π^d^	0.0050	0.0019	0.0050	0.0090	0.0050	0.0036	0.0053	0.0019
SD of Nucl. div.	0.0003	0.0006	0.0004	0.0018	0.0003	0.0008	0.0006	0.0006
Tajima’s D value^e^	−1.723	−1.863^f^	−1.542	−0.563	−1.560	−1.775	−1.859^f^	−1.782
dN/dS^g^	n.a.	n.a.	0.0485	n.a.	0.0603	n.a.	0.180	n.a.

a Total number of variable positions found relative to the PMCV reference isolate AL V-708.

b Number of sequence variants and sequence diversity were provided from the DnaSP v6 software’s resulting values of haplotypes (h) and haplotype (gene) diversity (Hd), respectively.

c Sequence diversity describes the uniqueness of a particular sequence variant in a given population, i.e. the probability that two sequences randomly sampled are different [varies between 0 (all sequences are equal) and 1 (all are different)].

d Nucleotide diversity, π, describes the average number of pairwise nucleotide differences between two randomly chosen sequences per position.

e Tajima’s D distinguishes between sequences evolving neutrally (Tajima’s D = 0) or under a non-neutral process (Tajimas D ± 0).

f Statistical significance of Tajima’s D value *P* < .05 (remaining D values not significant).

g Ratio of non-synonymous substitutions per non-synonymous site (dN) to the number of synonymous substitutions per synonymous site (dS), dN/dS, describes the strength and mode of natural selection acting on coding regions (dN/dS =1 indicates neutral selection).

nts—nucleotides. SD—standard deviation. n.a.—not applicable.

**Figure 3.**
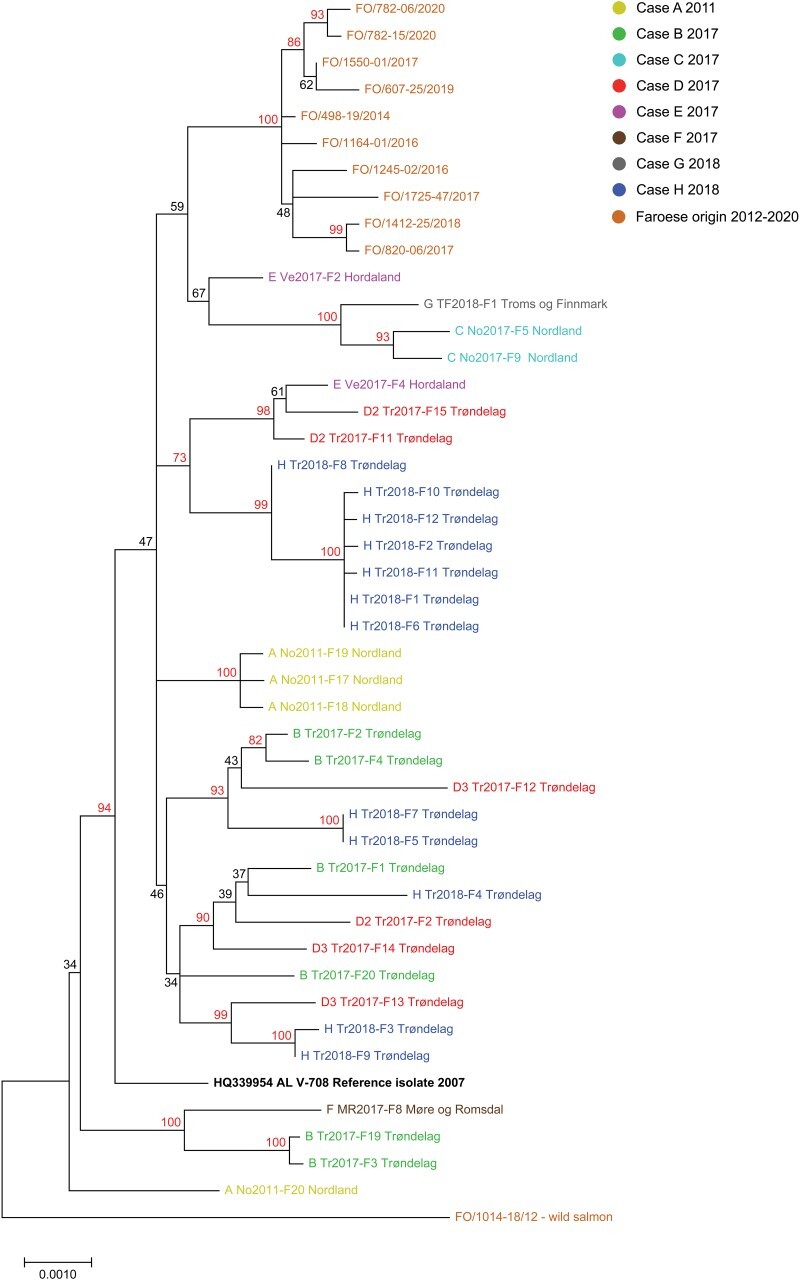
Phylogenetic analysis of 34 Norwegian and 10 Faroese PMCV complete and near-complete genome sequences from Atlantic salmon aquaculture sites experiencing CMS outbreaks or with suspected CMS. One near-complete genome sequence obtained from Faroese wild Atlantic salmon, sampled in 2012, and the Norwegian reference genome sequence AL V-708 from 2007 are also included. Norwegian sequences (Cases A–H) are color-coded by case origin and identified with the unique ID (“case” “two letter code for Norwegian county of origin” “year of sampling”-“fish number”’) followed by county of origin. Sequences from the Faroe Islands are all shown in light brown color and identified with the unique ID (FO/ “case number”-“fish number”/“year of sampling”). All bootstrap values 70 or above are shown in red color. The tree was rooted to the sequence from Faroese wild Atlantic salmon by default due to its high divergence towards the remaining 45 sequences. GenBank accession numbers are given in Supplementary Table S3.

**Figure 4.**
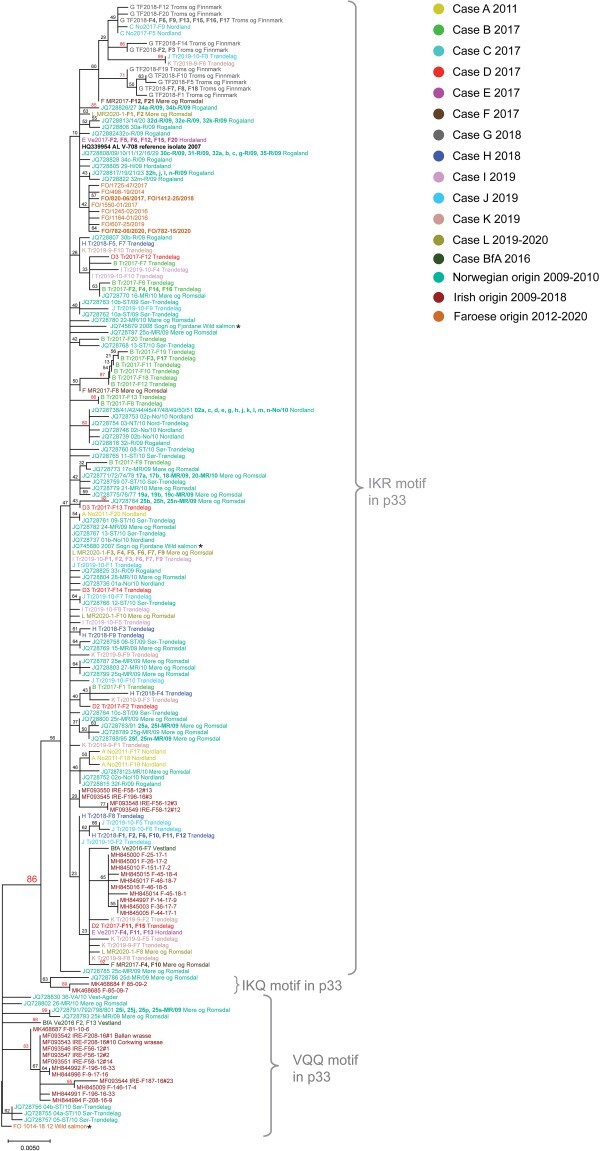
Phylogenetic analysis of 251 complete or near complete PMCV ORF3 sequences, including all ORF3 sequences obtained in the present study and sequences publicly available from Norway, the Faroe Islands, and Ireland. The ORF3 sequences extracted from the full-genome sequences and additional ORF3 sequences obtained from Norwegian Cases A–H are color-coded and identified with unique IDs as in Fig. 3, with extra color coding for Cases I–L and Bf A. From the three time points available from Case D, near-complete sequences for phylogeny were only available from sampling points D2 and D3. For Cases I–L, only sequences from the latest time point of the time course are included. Previously published Norwegian sequences from 2009 to 2010 and sequences from the Faroe Islands and Ireland are given as indicated with a single color, independent of case origin. These Norwegian sequences are identified by GenBank accession number and unique ID (“case no.” “fish ID by lower case letter”- “letter code for Norwegian county of origin”/year of sampling). Sequences from cases of which only one individual is included are not identified using fish ID by lower-case letter. Faroese sequences are identified with the unique ID (FO/“case number”-“fish number”/“year of sampling”) and Irish samples by GenBank accession number and unique ID including info on case (e.g “IRE-F58” or “F-25”), followed by year of sampling and fish ID (e.g. “#13” or “1”). The Norwegian PMCV reference genome sequence AL V-708 from 2007 is highlighted in black. All sequences originate from farmed Atlantic salmon unless otherwise stated. The ORF3 sequences from wild salmon are highlighted with asterisks. All Norwegian sequences are also provided with information on the county of origin. For display purposes, the tree has been manually compressed by showing identical Norwegian ORF3 sequences originating from the same case in the present study together on the same branch. For the remaining sequences from Norway, identical sequences are shown on the same branch only if obtained from the same county, with the accession numbers followed by an ID in respective order. Identical sequences from the Faroe Islands are also shown on the same branch regardless of origin. All bootstrap values 70 or above are shown in red color with the value separating the two main groups in the tree highlighted by a larger font size. Brackets are used to group sequences encoding the IKR, IKQ, or VQQ motifs in residues 84, 87, and 97 of the ORF3-encoded protein p33 (see text for details). The tree has been rooted against the wild salmon PMCV ORF3 sequence from the Faroe Islands, also for display purposes.

Our multiple sequence alignments and phylogeny indicated inter-host sequence variation of PMCV among the individuals in single cases (see sections below, including Figs 3 and 4). To compare diversity and evolutionary selection measures for the 35 full genomes representing various cases to full genomes originating from a single case, the above analyses were performed for the 12 genome sequences available from Case H as a separate subset (Supplementary Table S4). The analyses revealed relatively high inter-host sequence diversity for sequences originating in a single case, described by nine full-genome sequence variants, a sequence diversity of 0.939, and a nucleotide diversity of 0.0034. The variation affected all coding regions and the internal UTR^2−3^, while no variation was observed in the 5ʹ and 3ʹ end UTRs. Although the diversity measures were lower than those observed in the 35 genomes from multiple cases, similar trends across the different regions were indicated. None of Tajima’s Ds were given as statistically significant. Consistent with the overall 35 genome results, a low dN/dS ratio was seen for all three ORFs.

### Phylogenic analysis reveals a lack of consistent spatio-temporal clustering of PMCV genome consensus sequences

A phylogenetic analysis was performed on the new 34 complete or near-complete genome consensus sequences from Norway (Fig. 3). Ten near-complete genome sequences originating from nine CMS outbreaks in 2014–2020 at the Faroe Islands and one sequence from a wild salmon from 2012, were also included (details and GenBank accession numbers are available in Supplementary Table S3). Overall, the genome sequences are highly similar. All genomes from the Faroese CMS cases form a monophyletic cluster, separate from the Norwegian sequences with high bootstrap support. Only the Faroese wild salmon sequence is located outside of this cluster. The tree also displays several internal nodes with high bootstrap support, which include both the clustering of Norwegian sequences originating from single cases as well as geographically distant cases (Fig. 3). Notably, seven of the twelve consensus sequences from Case H group together with high bootstrap support, while the remaining five cluster together with sequences from Cases B and D, also with high bootstrap support. Most Norwegian sequences were sampled over a limited period in 2017-2018. However, sequences from the only case significantly separated in time (Case A, 2011) are distinct from all of the other sequences in the tree. This is also the case of the reference sequence AL V-708 from 2007, the earliest sequence available, and the sequence from the Faroese wild salmon from 2012. In summary, the phylogenic analyses show a spatio-temporal clustering based on geographic origin by country and sequences with origin separated by a long-term period, but no consistent spatio-temporal clustering at a higher resolution. The PMCV genome consensus sequences are highly similar but with some case-to-case variability and even inter-host sequence variability within the single cases.

To identify PMCV variants with potentially varying virulence observed in specific cases or associated with defined clusters seen from phylogeny, we analyzed the relationship between specific PMCV variants, heart lesion scores, and PMCV RNA levels, in a subset where the relevant data were available per individual (see Supplementary Material S1). No specific virus variants could be associated with high or low virulence.

### Phylogenetic analysis of PMCV ORF3 sequences reveals that Norwegian, Faroese, and Irish variants group separately

Since a large dataset of PMCV ORF3 sequences are publicly available from previous sequencing studies, we included an additional focus on this ORF by Sanger sequencing of PCR amplicons of additional samples from the cases described in this study. Phylogenetic analyses were performed on the sequences from these additional samples, ORF3 extracted from the new Norwegian and Faroese full-genome sequences, and previously published sequences.

In total, the analysis included PMCV ORF3 sequences from 87 Norwegian, Faroese, or Irish cases spanning the years 2009–2020, in addition to sequences from wild Norwegian and Faroese salmon and Irish corkwing wrasse and Ballan wrasse (details and GenBank accession numbers are available in Supplementary Table S3). The resulting phylogenetic tree shows that a high number of sequences are identical, or nearly identical, with low bootstrap support for most branches. Still, the ML tree generated can be partitioned into one large monophyletic and a smaller paraphyletic cluster with high bootstrap support (Fig. 4). Most of the Norwegian sequences, some of the Irish and all Faroese sequences of aquaculture origin are contained within the larger cluster. The sequence from wild Faroese salmon, remaining Irish sequences, a few Norwegian sequences from 2009 to 2010, and one broodfish sequence found in two individuals from 2016 are found within the smaller paraphyletic cluster. The Irish and Faroese sequences group separately but with low bootstrap support in both the monophyletic and paraphyletic cluster. Although most branches in the tree have low bootstrap support, some sequences from Norway originating from individual cases form smaller groups with high bootstrap support. The grouping of sequences, or lack thereof, in the ORF3 tree supports the result from phylogenetic analysis of the full-genome sequences (Fig. 3), i.e. PMCV variants do mostly group according to geographical origin by country, but there is a lack of consistency in spatio-temporal clustering.

Phylogenetic analyses on PMCV ORF1 and ORF2 were also performed, using ORFs extracted from the Norwegian and Faroese complete genomes available from the present work and for ORF1 also including Norwegian sequences from GenBank (Supplementary Fig. S2a and b). As none or only partial ORF1 or ORF2 sequences are available from Ireland, Irish sequences were not included in the trees. The ORF1 and ORF2 trees display grouping patterns similar to the phylogenetic trees generated from the full genomes and ORF3 (Supplementary Fig. S2).

### The inter-host PMCV sequence variation in a case may be a reflection of the intra-host sequence variation

**Figure 5.**
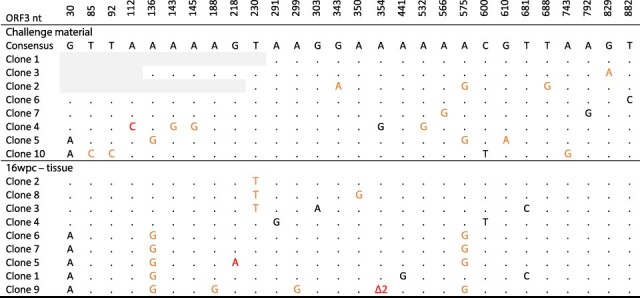
Diverging nucleotides observed between ORF3 amplicon clones from a cell culture preparation of PMCV challenge material and ORF3 amplicon clones originating from heart tissue of one Atlantic salmon parr at 16  wpc, infected using the challenge material. For comparative presentation purposes a consensus of the most prevalent nucleotides found in the challenge material amplicon clones are given on top. Nucleotides (nts) in the clone sequences deviating from this consensus are indicated, with a dot representing no difference. Black nts—synonymous mutation, orange nts—non-synonymous mutation, red nts—mutation resulting in stop codon or codon frame shift, Δ2—deletion of two adenines within a stretch of six adenines in positions 349–354. Grey background—no information due to low-quality of sequencing chromatograms.

The multiple PMCV consensus sequence variants obtained from individual hosts in single cases may reflect high intra-host diversity, where the consensus sequence from each individual represents the master or dominating variant in that individual. Examination of chromatograms from Sanger sequencing of ORF3 occasionally revealed double peaks at some positions (i.e. mixed nucleotides), suggesting the presence of more than one dominating variant in these individuals. Intra-host PMCV sequence diversity was then investigated further through two approaches. First, single plasmid clones of ORF3 were obtained from PCR amplicons prepared from both PMCV infection challenge material of cell culture origin and from heart tissue of one fish sampled at 16 wpc after infection using the challenge material (Table 1, Ex A). ORF3 sequencing from multiple plasmid clones, each representing a single PCR amplicon, revealed variation within the ORF3 amplicons sequenced from both the challenge material and the infected fish heart tissue (Fig. 5). Only two of the nine clones from infected tissue had identical ORF3 sequences. However, unique nucleotide differences were found in all clones in the remaining sequences. Both synonymous and non-synonymous mutations were found by comparison with the PMCV ORF3 AL V-708 reference sequence. Examples of mutations introducing stop codons or smaller deletions shifting the reading frame were also seen.

**Figure 6.**
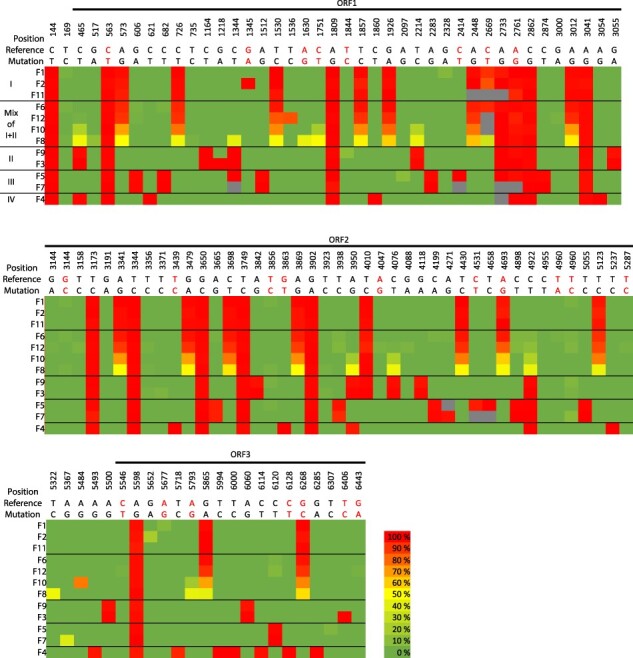
Intra-host single-nucleotide variants (iSNVs) with variant frequencies identified from PMCV sequence reads from RNA seq obtained from the 12 individuals of Case H. The nucleotide variants, and their positions in the genome with ORFs indicated, are shown relative to the PMCV reference AL V-708 reference sequence. Nucleotides colored in red indicate non-synonymous variants. Varying nucleotides in <2% of the reads are excluded. Frequencies of iSNVs are shown per individual as a colored heat map (green 0–2%, yellow 50%, red 100%). Grey color indicates no sequence information. Black lines separate the samples into four groups (I–IV) based on similarity of iSNVs. F6, F8, F10, and F12 are characterized by a mix of iSNVs described by groups I and II in varying frequencies.

The second approach focused on iSNV analysis of the RNA seq data from the 12 individual samples of Case H. For each sample data set, the reference-based assemblies were analyzed for single nucleotide variations at each position (Fig. 6). As revealed by the phylogenetic tree (Fig. 3), the sequences were separated into four clusters. However, the iSNV analyses from each sample show numerous unique positions at low frequencies differing from the reference genome and the sample consensus sequences (only frequencies of 2% and higher are included). Interestingly, the data show that some individuals have a set of iSNVs that may indicate a mix of two abundant genome sequence variants. This is exemplified by fish individual F8, which includes an approximate 50/50 mix of iSNVs/mutations represented by F1, F2, and F11 mixed with variants of F3 and F9. A similar mix is also apparent for F10, F12, and F6, but the ratio is more skewed in ascending frequency, respectively, towards the variants seen in F1, F2, and F11. In the phylogenetic tree (Fig. 3), the consensus sequences from F1, F2, F11, F10, F12, and F8 form a group with high bootstrap support. However, those from F9 and F3, F5 and F7, and F4, cluster in three separate groups, of which all additionally includes consensus sequences from Case B and D and is supported by high bootstrap values. The sequences from Case B, D, and H in each of these three groups have common mutations that are unique to the group. However, Case B and D sequences also have unique mutations that are not found in either Case H consensus sequences or as iSNVs of low frequency.

### The progress of PMCV replication and severity of CMS coincide with virus diversity under field conditions

**Figure 7.**
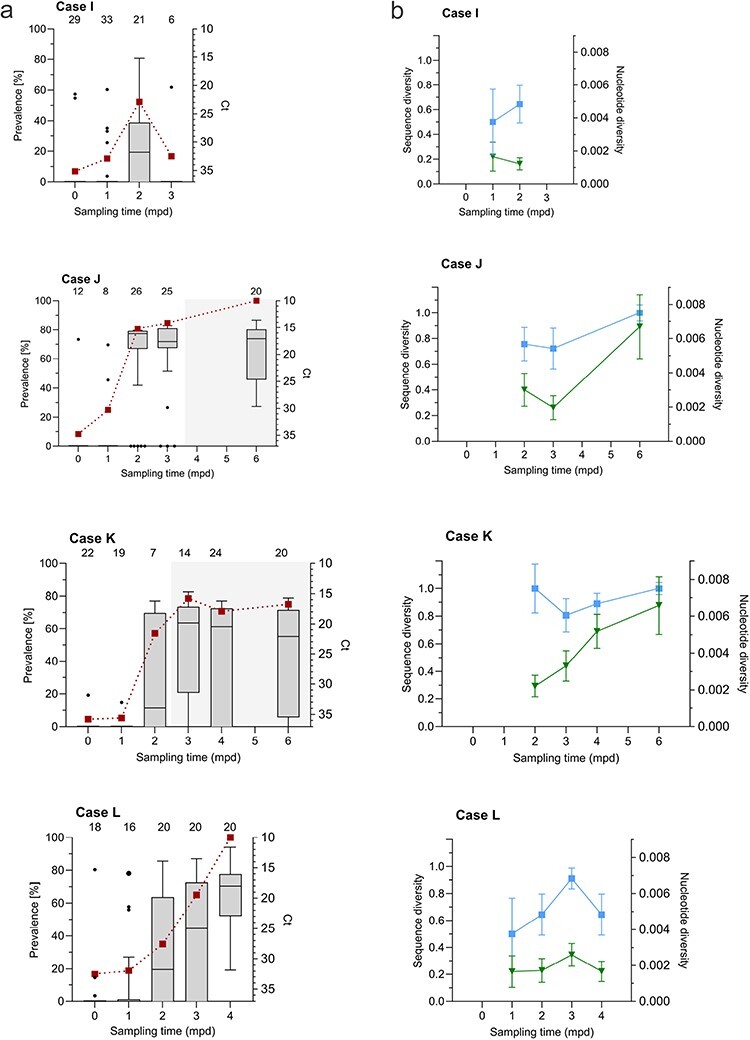
Characteristics of PMCV infection progress and ORF3 diversity over time post first virus detection in field populations (Cases I–L). (a) Prevalence of fish positive for PMCV RNA of the total number tested (*n* = 6–33 as indicated on top of diagrams) following monthly real-time PCR screening of the study populations in Cases I–L from primary detection (0  mpd; red squares, left axis). PMCV RNA levels per individual are indicated by Ct values (right axis). Negative samples are included, all set to Ct 37 (the cut-off Ct defining a negative result). The Ct data are presented as box plots representing the interquartile range (middle 50% of the data) with a median line. Whiskers are drawn from minimum to maximum. Outliers are indicated by dots beyond either whisker. The larger dot symbol (Case L, 1 mpd) represents two samples differing by 0.5 Ct. Sampling time points are given as mpd. Onset and period including mortality in Cases J and K is indicated by grey shade in the diagram. (b) Sequence diversities (blue squares, left axis) and nucleotide diversities (π, green triangles, right axis) calculated for ORF3. Sequence diversity describes the uniqueness of a particular sequence in a given population, i.e. the probability that two sequences randomly sampled are different. Nucleotide diversity (π) represents the average number of pairwise nucleotide differences between two randomly chosen sequences per position. The two diversity measures are only calculated for time points where a minimum of four ORF3 sequences are available (*n* = 4–10). Sampling time points are given as mpd.

Nucleic acid samples were made available from four additional field study populations (Cases I–L) aimed at studying diversity over time. These populations had been kept at different aquaculture sites, and a selection of the fish (*n* = 6–33, by mean 19 per time point/case) had been sampled and screened for PMCV monthly from sea transfer until slaughter (a period of up to 13–18 months). In short, PMCV was first detected approximately one year post-sea transfer, with a low prevalence among the tested fish and low levels of PMCV RNA per individual in all four study populations at first detection. At later time points, virus replication showed different profiles, seen as differences in number of positive fish and levels of PMCV RNA per individual (Fig. 7a).

Sanger sequencing of ORF3 showed that inter-host sequence variation was apparent for Cases I–L at all time points, consistent with our previous results from Cases A–H. Still, the diversity measures varied between the cases and with time (Fig. 7b).

Cases J and K showed an abrupt increase in PMCV positives and virus levels per fish for the first months after primary detection. Increasing mortalities in the following months resulted in high cumulative mortalities in these populations, i.e. 5% and 20%, respectively. For Case J, there was a significant linear trend for increase in nucleotide (*P* < 0.0001) and sequence diversity (*P* =0 .0007) between the two first samplings and the last sampling (Fig. 7b). For Case K, there is no difference in sequence diversity between any of the time points (Fig. 7b), while there is an overall significant linear trend for increase in nucleotide diversity over time (*P* < 0.0001, Fig. 7b).

Case L showed a gradual rise in PMCV RNA per individual/population over the same period with lower cumulative mortality (1.5%). There was a significant linear trend for increased sequence diversity over time (*P* < 0.0001, Fig. 7b); however, with a drop at the last sampling time. There is no linear trend for increase in nucleotide diversity over time (Fig. 7b, *P* = 0.624).

Case I deviated from the other cases, as the number of PMCV RNA-positive individuals was generally low at all time points. Only a minor increase in the number of positive fish and viral RNA levels was seen over the 2 months following primary detection before the prevalence and viral RNA levels decreased again, correlating with no CMS diagnosis and no significant mortality. Due to the low number of PMCV RNA positive samples, ORF3 sequences were only available for calculations of diversity measures from two time points. Still, the resulting values were generally lower than for Cases J and K but comparable to Case L values at similar time points.

As mentioned earlier, virus diversity in Cases J and K increased with time, and this correlated with the increased virus replication and observed CMS-induced mortality for these two cases.

### Genetic diversity is low and stable over time under experimental challenge conditions, in contrast to the diversity observed under field conditions

**Figure 8.**
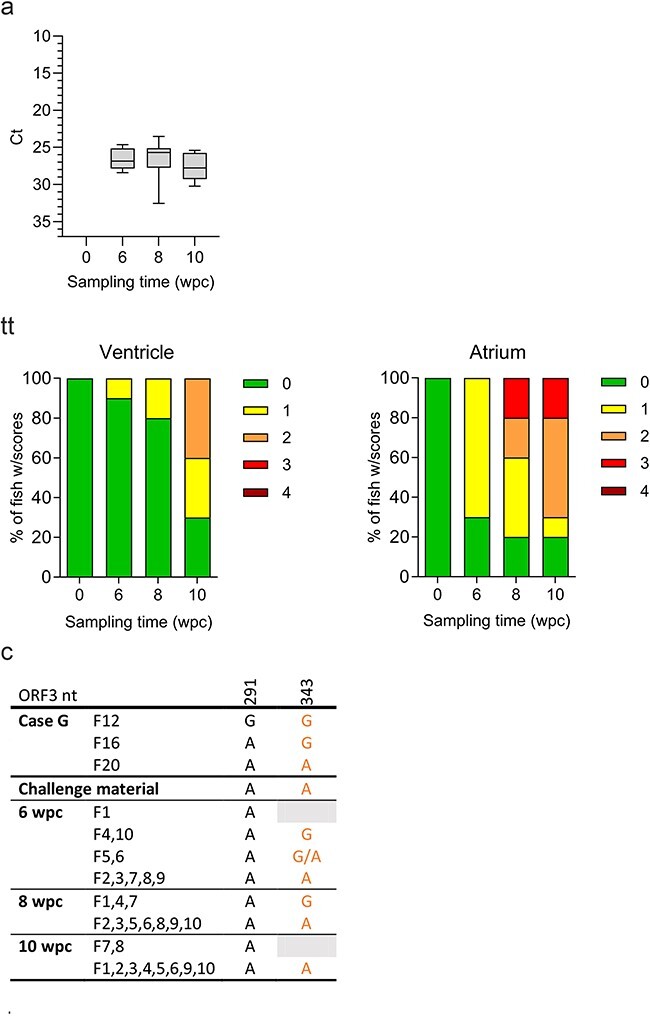
Characteristics of PMCV infection progress and diversity over time post-challenge in an experimental challenge (Ex B). (a) Levels of PMCV RNA indicated by Ct values for all sampling time points in the wpc in heart tissue. The Ct data are presented as box plots representing the interquartile range (middle 50% of the data) with a median line. Whiskers are drawn from minimum to maximum. (b) Distribution of histopathology scores of the atrium and ventricular cardiac compartments among the individuals for all sampling time points, with scoring from 0 (no lesions observed) to 4 (severe lesions). No fish with a score of 4 were found at any time point in any cardiac compartments. (c) Comparison of available PMCV ORF3 sequences from the field samples Case G F12, 16 and 20 pooled for the challenge material, the resulting challenge material, and from challenged fish from all time points with position and nucleotide present of the only two positions of a total of 909 in ORF3 with observed diversity. Two nucleotides separated by a slash indicate that they were both found in significant amounts among the reads from RNA seq. Black nts—synonymous mutation, orange nts—non-synonymous mutation, grey background—RNA seq data resulted in low or no coverage. RNA seq of the challenge material and all heart tissue samples produced partial genomic sequences covering more than ORF3.

We also included an alternative approach to analyze PMCV sequence diversity over time *in vivo*, based on samples from Atlantic salmon parr infected under experimental conditions. The challenge inoculum was prepared from a pooled homogenate from heart tissue samples from Case G and fish were injected individually. Heart tissue was subsequently sampled in ten individuals at 6, 8, and 10 wpc. Real-time PCR analyses confirmed PMCV infection in all sampled individuals, with no significant differences in viral RNA loads at any time point. Lesions characteristic of CMS were found in the heart, with increasing severity over time (Fig. 8a and b). RNA from the challenge material and all heart tissue samples were subjected to RNA seq. Unfortunately, the sequencing did not produce sufficient depth and coverage to obtain full-length genomic sequences for all samples. Consequently, pairwise sequence alignments and comparative analyses were based solely on ORF3 (Fig. 8c). Contradictory to the results from the field cases, the analyses of these sequences indicated an in general very low genetic diversity of PMCV among the individuals and provided no indications of increasing diversity over the 10-week infection period. Two variable positions were identified from the ORF3 sequences from the three individual heart tissue samples from Case G (nucleotides 291 and 343). For nucleotide 291, the A in two of the three samples was apparent in the resulting inoculum and all infected individuals. For nucleotide 343, the A present in one of the three samples was seen in the inoculum, but still resulted in varying presence of the two nucleotides (A and G) among the infected individuals. Pairwise sequence alignment including additional partial PMCV genomic sequences from RNA sequencing supports these results and can be found in Supplementary Fig. S3.

### The PMCV p33 variant identical to the reference AL V-708 is frequent among individuals across cases, combined with minor numbers of case-specific variants

All ORF3 consensus sequences generated in this study were translated to p33 protein sequences and used in multiple sequence alignments, including p33 from AL V-708 isolate as reference (Fig. 9). Interestingly, p33 equal to the AL V-708 reference variant was found in multiple individuals from most field cases. In each case, this p33 variant was usually found in concert with several other less prevalent variants among the individuals. These less prevalent variants found per case usually appeared with amino acid substitutions specific to the case, with a few exceptions. One remarkable exception is the E_243_Q substitution, observed from several individuals across nine cases.

**Figure 9.**
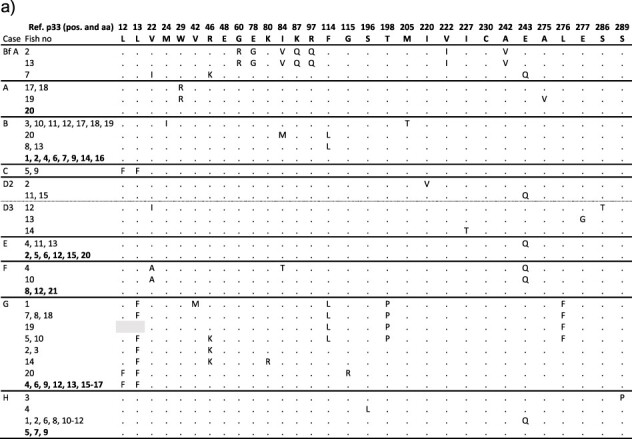
PMCV p33 protein sequence variants from Cases A–L and broodfish Case Bf A. Amino acid substitutions in the PMCV ORF3-encoded p33 shown relative to the AL-V 708 reference p33 sequence with residue numbers and amino acid in reference shown on top. Fish individuals are identified by fish number and those with identical p33 sequences are shown on the same row. Fish individuals with a p33 sequence identical to the reference sequence are highlighted in bold. Grey background indicates that sequence information was not obtained. (a) Cases Bf A and A–H. (b) Cases I–L, including p33 sequences from each sampling time point (mpd). A highly divergent variant found in single fish from both Cases J and K (see Fig. 4, J Tr2019-10-F8 and K Tr2019-9-F6, uppermost part of tree) is marked by an asterisk.

**Figure 9.**
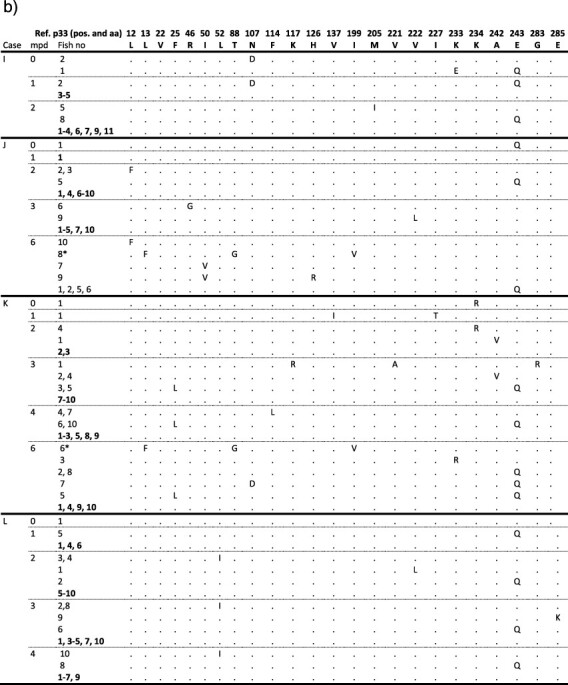
continued

The combination of I_84_V, K_87_Q, and R_97_Q substitutions, described as an IKR motif substituted with a VQQ motif (Wiik-Nielsen et al. 2012), is also worth noting. Among the recent Norwegian p33 variants from the present study, the VQQ motif was only found in p33 of a subset of the individuals of one case from Norway, i.e. the broodfish Case Bf A from 2016. It was also found in the Faroese wild Atlantic salmon sequence (from 2012). In the phylogenetic tree generated from all currently available ORF3 nucleotide sequences (Fig. 4), the large monophyletic and small paraphyletic clusters represent the IKR and VQQ motifs of the translated protein sequences, respectively. An intermediate of the two, the IKQ motif found in one Norwegian salmon and two from the same case in Ireland, all from 2009, form a separate group within the larger monophyletic cluster, although with poor bootstrap support.

For the field cases studied over time (Cases I–L), no p33 variants or specific amino acid substitutions changed prevalence over the study time course in any of the four cases (Fig. 9b). Still, for Cases J and K the highly prevalent p33 reference variant was indicated to be less prevalent at late time points while the number of additional variants of p33 increased. This supports that the increase in genetic diversity seen over time for PMCV ORF3 from these populations is also resulting in an increase in protein diversity. These results also show general inter-host variation of protein sequences (Fig. 9), supported by our results from phylogeny and detailed analyses of nucleotide sequences (Figs 3 and 4), as well as by previous studies (Wiik-Nielsen et al. 2012).

The phylogenetic tree generated from ORF3 sequences includes only sequences from the last sampling point available from Cases I–L. In the tree, a highly divergent variant (9 divergent nucleotides (1.0%) relative to reference ORF3) was found in a single fish from both Cases J and K (Fig. 4, J Tr2019-10-F8 and K Tr2019-9-F6, uppermost part of tree). The number of divergent nucleotides to remaining variants from both cases was 10–15 divergent nucleotides (1.1–1.7%). This shared ORF3 variant is also seen as a shared variant in these two cases, from the translated p33 amino acid sequences, in addition to the highly prevalent p33 reference variant (Fig. 9b, Case J/6 months post-detection (mpd)/F8, and Case K/6 mpd/F6). It is worth noting that the fish in the study populations in these two cases are of common origin, and the aquaculture sites are located close together in the same fjord. The first detection of PMCV was 8–9 months post-sea transfer for both, and both experienced high CMS-related mortality.

### PMCV produces defective viral genomes

**Figure 10.**
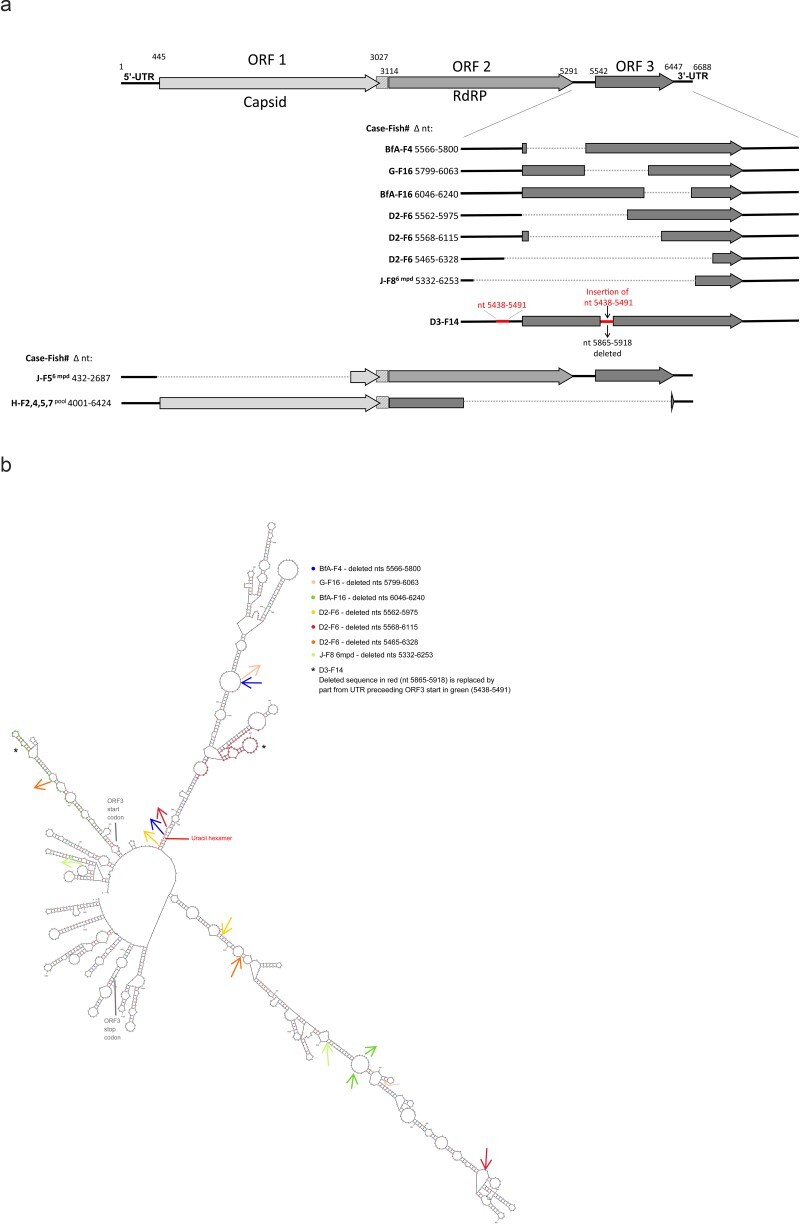
Deletions identified in PMCV RNA and their relation to predicted RNA secondary structure. (a) Schematic presentation of the deletions found in the PMCV genome from a selection of infected tissue samples from field cases. Deleted nucleotides (Δ nts) are indicated by positions relative to the 5ʹ end of the genome. One deletion-insertion event resulting in 54 nts of UTR^2−3^ replacing a deletion of equal size in ORF3 is also shown. (b) Predicted secondary structure of ORF3 RNA, including upstream UTR^2−3^ and downstream 3ʹUTR. Color-coded arrows indicate where each deletion is initiated (arrow facing out) and ends (arrow facing in). Hexamer of uracils where deletions are initiated are indicated by red letters. Nucleotides representing a likely deletion-insertion event are indicated in red (deleted) and green (inserted) shades.

During the Sanger sequencing studies of ORF3 from various CMS field cases, we sporadically discovered PCR products of shorter lengths than the 1.2 kb expected product. Sanger sequencing of these shorter products confirmed them to be primer-specific amplifications but characterized by deletions. These deletions varied in sizes (54–864 nt in length), and position in the ORF3 amplicon (Fig. 10a). One likely deletion-insertion event, characterized by a deletion of 54 nucleotides in ORF3 replaced by 54 nucleotides from the UTR preceding the ORF3 (Fig. 10a), was also identified. We then attempted to amplify larger parts of the genome (see amplicon overview in Supplementary Table S2) and found, similarly, shorter products characterized by deletions (Fig. 10a). Deletions were found related to both the ORFs and the UTRs. Since secondary structures are suggested to initiate deletions (Rowe et al. 1998, Petterson et al. 2016, Levi et al. 2024), we mapped the deletions identified in ORF3 and surrounding UTRs to predicted RNA secondary structures. Most deletions were initiated and ended in open loops in the predicted RNA structure, and alternatively, deletions were also initiated within a hexamer of uracils (Fig. 10b).

## Discussion

Sequence analyses from multiple sequence alignments and phylogenetic studies reveal that the PMCV genome is highly conserved, with consensus sequence variants consistently separated by country of origin. However, we find no consistent spatio-temporal linkage between different PMCV variants sampled within Norway, confirming findings from a previous study based on concatenated ORF1–ORF3 sequences (Wiik-Nielsen et al. 2012).

Moreover, with the now more extensive full-genome data set available, we observed that single CMS outbreaks include several PMCV variants among infected individuals. This observation is also supported by the extended ORF3 sequence dataset produced here and by the analyses of ORF1 and ORF2, confirming results indicated from previous studies (Wiik-Nielsen et al. 2012, Tighe et al. 2019), now at the full-genome level and by higher resolution findings. Sequences from individual cases grouped into one or several clusters. However, some sequences originating from one case also grouped with sequences from another case with high bootstrap support. Grouping sequences from different cases were not 100% identical over the full genome, but the number of positions that differed was low. However, in the phylogenetic tree based on ORF3, sequences originating from different cases separated in time by nearly a decade could be 100% identical. The fact that some sequences from different cases are identical or almost identical at the consensus sequence level, with no apparent spatio-temporal linkage between them, probably excludes genetic drift as a driver for this differential clustering.

The differential clustering of sequences originating from the same case may be explained by viral population diversity due to favoring one genome master sequence variant over another from a small intra-host pool of genome sequences. Based on our results from full-genome sequencing and the more comprehensive set of ORF3 sequences, we hypothesize that a few similar genome master sequence variants, randomly occurring from error-prone RdRp activity and/or selected during transmission, are associated with Norwegian CMS outbreaks. We also observed that several nucleotide divergencies were unique to PMCV from a specific case or even from single individuals within a case. In addition, the time course studies of Cases J and K indicated a time-dependent increase in PMCV sequence diversity, i.e. diversity increased with the prevalence of the virus in the population and viral RNA levels in individual fish.

These results suggest that inter-host sequence diversity may reflect the intra-host sequence diversity. High inter-host sequence diversity following passaging in a population or low intra-host sequence diversity at the time of or early in infection, with a subsequent increase in diversity, are both phenomena typically associated with tight bottleneck events where only small subpopulations of the virus are transmitted to the next host (Sheldon et al. 2014, Yu et al. 2018, Kadoya et al. 2020, Amato et al. 2022). The bottleneck effect provides sequences originally in the minority a chance to expand in a new sequence space made available in the naïve host (Domingo 2019, Kadoya et al. 2020).

We speculate that PMCV transmission occurs through bottleneck events where only a portion of the original sequence population is transmitted to the naïve host(s). The new sequence space made available is then free to be occupied by new genome variants arising from low-fidelity viral genome replication, with stochastic host factors likely playing a dominant role in the directional expansion of the mutant swarm and its composition. The grouping pattern observed for the PMCV consensus sequences in the phylogenetic analyses, the uniqueness of mutations observed between individuals in our consensus sequence data sets, and the time-dependent increase in sequence diversity in the timecourse study all support this hypothesis. Such expansions of intra-host genetic diversity have been associated with increased viral fitness (virulence and replicative efficiency) and vice versa (Vignuzzi et al. 2006, Ciota et al. 2007, Sheldon et al. 2014, Kadoya et al. 2020). Whether PMCV master sequence variants are transmitted from within the infective subpopulation (and may undergo some genetic drift) and/or selected during the early phases of viral replication in newly infected hosts is not known. It should also be mentioned that co-infection or superinfection following horizontal transmission from nearby infected sites within a short time frame should not be excluded and would be difficult to detect in our studies.

Contradictory to the apparent trend of general virus diversity per case (Cases A–L) and change in virus diversity with time under field conditions (Cases J–L), the time course study performed under experimental conditions showed a very low genetic diversity across the individuals. It did not suggest selection or expansion of genome sequence diversity from the inoculum and throughout the study period of 10 wpc. There might be several reasons for this discrepancy. First, in the experimental challenge trial, fish were infected simultaneously through an artificial route, i.e. i.p. injection with tissue homogenate from PMCV-infected fish. This bypasses potential bottlenecks experienced by the natural route of infection through horizontal transmission and within-host anatomical barriers represented by pathways to target organ, tissue, and/or cell type (Weaver et al. 2021, Amato et al. 2022). Consequently, the PMCV infection under experimental conditions was initiated without an early selection of a subpopulation of virus variants, which would likely occur during the natural route of infection. Additionally, the number of fish in the experimental trial is low compared to the number typically found in a field cage, which may contain up to 200 000 fish. Therefore, it cannot be excluded that the lack of increased PMCV genome sequence diversity during the trial is associated with factors related to founder effects (Weaver et al. 2021, Amato et al. 2022). In the field, CMS outbreaks will likely experience more complex infection dynamics in a much larger population than in an experimental challenge trial, where all animals are infected simultaneously. As the disease outbreak progresses in a field cage (or across multiple cages at a site), more hosts will get infected, and naïve hosts will inevitably become exposed to viral subpopulations shredded by numerous hosts of high mobility. The increase in viral loads may also influence the size of the bottleneck and affect the size and composition of the infecting subpopulation, and hence the sequence space made available in the new host (Zwart et al. 2011, Tao et al. 2014, Varble et al. 2014). Therefore, in addition to factors associated with the host and environment, the size of the fish population and the rate of infection spread in the population after first introduction are both likely to influence the development of genetic diversity and the selection of a master sequence or sequences. Future studies on PMCV infection dynamics and bottlenecks could benefit from being conducted at conditions that mimic the natural route of infection, such as infection by bath challenge or cohabitant shedder fish. However, experimental challenge trials under controlled host and environmental conditions will never accurately replicate the varying host and conditions in the field.

Although our results mainly describe inter-host sequence diversity of PMCV sequences originating from single cases and sampling time points, the consensus PMCV sequences *per case* primarily consist of divergent nucleotides unique to each case. This supports the hypothesis that only a few PMCV variants are transmitted to naïve hosts. This likely includes variants with high similarity to the PMCV AL V-708 reference variant, which we have shown to have an almost ubiquitous presence in all cases based on the ORF3 sequences and its translated p33. Finally, host factors such as genetics and immune responses are also very likely to influence the direction of the sequence expansion in each individual. Information on the salmon breed has only been available for Cases I–L, and the fish populations of cases J and K have been identified as being of a single breed. As for all cases studied, unique mutations were also found per case for these, but interestingly, ORF3 sequences from one single individual sampled at the latest time point in each of Cases J and K were found to be 100% identical to each other (Cases J F8 and K F6, both 6 mpd). In addition to the common origin of the two fish populations, the locations of these two cases were within a close geographical distance of only a few kilometers at sea. Together, this may suggest that either genetics of the host and/or environmental factors may have played a role in producing these two identical ORF3 sequences.

For all three PMCV ORFs, we found low dN/dS ratios, suggesting purifying selection with the removal of non-synonymous mutations over time and/or due to large population sizes (Holmes 2009, Lin et al. 2019). Low inter-host dN/dS ratios are not uncommon in RNA viruses, where negative selection is much more prevalent than positive selection (Holmes 2009). However, ORF3 displayed a comparatively higher dN/dS value, indicating that a higher number of positions in this gene may be under positive selection compared to ORF1 and ORF2, which affects the resulting gene-wide average dN/dS value. The negative Tajima’s D values obtained for all three genes and the UTRs may indicate selective sweeps and/or recent population expansions, bottlenecks, or, as also indicated by the low dN/dS values, purifying selection (Tajima 1989). However, in contrast to the dN/dS ratio for ORF3, the lower Tajima’s D value for ORF3 compared to the two other genes may suggest increased purifying selection specifically for this gene.

In the field, the severity of outbreaks can vary, and while PMCV has been detected in individual fish, it does not always lead to clinical CMS in the broader population (Svendsen et al. 2019, Case I in this study). Studies have examined risk factors associated with the development of clinical CMS in natural settings (Bang Jensen et al. 2013), though specific virulence factors remain unidentified. This study, however, highlights some notable non-synonymous and synonymous mutations, or mutation motifs, with potential relevance to virulence and fitness. One such mutation is the E_243_Q substitution in p33, which appears frequently in our dataset across several Norwegian cases, albeit only in certain individuals within each case. Interestingly, this prominent non-synonymous mutation had not been observed in ORF3 sequences prior to this study (Wiik-Nielsen et al. 2012, Tighe et al. 2019). This suggests that the E_243_Q substitution may have emerged recently and could be specific to Norwegian PMCV variants. The non-synonymous mutations resulting in amino acid changes between the IKR and VQQ motifs at residues 84, 87, and 97 of p33 are also noteworthy. In our present Norwegian dataset including sequences from years 2016 to 2020, the VQQ motif has been present only in a few broodstock fish in Case Bf A in 2016. However, this motif was prevalent in Norway from 2009 to 2010 and in Ireland up to 2017. Apart from being found in one wild salmon in 2012, it has not been observed in Faroese aquaculture sequences from 2014 to 2020, suggesting that the VQQ motif may have undergone negative selection. The potential influence of the p33 E_243_Q substitution or the shift between IKR and VQQ motifs on PMCV virulence and disease severity has not yet been studied. Notably, the VQQ motif often accompanies the V_222_I substitution in Norwegian PMCV variants and Faroese wild salmon, while the V_222_T substitution appears in Irish variants, each with additional case-specific substitutions. So far, both IKR and VQQ motifs have been found in CMS outbreaks and individual fish with CMS characteristic lesions in the heart, as well as in fish not associated with CMS (this study, Wiik-Nielsen et al. 2012, Scholz et al. 2017, Tighe et al. 2019).

The E_243_Q substitution is consistently found concurrently with the synonymous mutations C_726_T and A_3012_G (ORF1) and C_3698_T (ORF2) in all available full-length genomic sequences (described in Fig. 2). Another example of concurrent non-synonymous and synonymous mutations is the highly prevalent non-synonymous mutation C_563_T, resulting in the amino acid change A_40_V in the capsid, along with the concurrent presence of synonymous mutations G_3650_C and G_3902_A (ORF2). Similar concurrent non-synonymous and/or synonymous mutations are observed in ORFs and UTRs, albeit with low prevalence.

Also worth noting are the two mutations, A_3041_G (in link^1−2^, synonymous if coding for a capsid-RdRp linker) and A_5598_G (ORF3, synonymous). These mutations are highly prevalent in Norwegian cases and are unique to Norwegian PMCV variants, as the two mutations were not found in any of the 43 Faroese PMCV sequences recently published, except for the single sequence from Faroese wild Atlantic salmon and two from farmed salmon. A phylogenetic analysis including additional Faroese PMCV sequences confirms these three to be outliers to the Faroese monophyletic cluster (Dahl MM, Christiansen DH, personal communication, unpublished data). Data are unavailable for these parts of the genome in sequences of Irish origin.

Virulence and virus fitness are multifactorial and may involve functional properties associated with single, if not all, virally encoded proteins. There is also growing evidence that synonymous mutations in pathogens can contribute to virulence and fitness by influencing the efficiency of transcription and translation by altering codon preferences, affecting transcription initiation related to promotor sites, causing ribosomal pausing, and influencing RNA structure and stability (Cuevas et al. 2012, Bailey et al. 2021).

In addition to the described diversity through non-synonymous and synonymous mutations, our results reveal that PMCV replication generates viral RNA with various deletions, i.e. producing defective viral genomes (DVGs), as previously also shown during infection by viruses from most virus families (Nagy and Simon 1997, Pathak and Nagy 2009, Markussen et al. 2013, Petterson et al. 2016, Vignuzzi and López 2019, Zhou et al. 2023). Mapping the observed deletions on a predicted RNA secondary structure indicated that the mechanism behind this may be related to RNA secondary structures (open loops) or uracil-rich stretches that encourage copy-choice recombination by template switching during genome replication or transcription, as also indicated for other viruses (Nagy and Bujarski 1993, Havelda et al. 1997, Rowe et al. 1998, Pathak and Nagy 2009, Petterson et al. 2016, Levi et al. 2024). Specific RNA structures or uracil-rich regions may cause the RdRp to pause, leading to its temporary release from the template while still attached to the nascent RNA strand. The RdRp may then rebind at a nearby position, either across the same open loop or to a neighboring loop as defined by the 3D structure environment of the RNA. DVGs are most commonly characterized by deletions of variable sizes, but insertion and even point mutations and hypermutations have been described in all significant viral families. In addition to the deletions detected in this study, the observed deletion-insertion event replacing a part of ORF3 with a part of UTR^2−3^, and the single mutations found or combinations thereof may result in DVGs. Like other viruses, DVGs generated from PMCV replication may play an essential role in virulence and as a determinant of pathogenesis by modulating the replication, influencing disease outcomes, triggering immune responses, or promoting virus persistence following infection (Rezelj et al. 2018, Vignuzzi and López 2019).

Genomic factors affecting PMCV virulence and CMS disease severity seem complex and have yet to be identified, characterized, and fully understood. It is likely that a better understanding of the functional characteristics associated with each of the three PMCV-encoded proteins, including any potential functional linkage between the proteins, is needed to identify putative virulence markers associated with specific amino acid residues, if present. Additionally, the synonymous mutations are intriguing due to their potential impact on the efficiency related to replication, such as transcription and translation, and as factors affecting the generation of DVGs and modulation of subsequent effects of DVG formation. Cell culture systems that support PMCV growth must be developed to enable the study of evolving genetic diversity and/or selection, and also the presence and development of DVGs *in vitro* over time under controlled conditions.

## Supplementary Material

veae097_Supp

## Data Availability

The raw sequence data from RNA seq has been submitted to the NCBI Sequence Read Archive (SRA) database (https://www.ncbi.nlm.nih.gov/sra) with BioProject ID PRJNA1064402. The data describing PMCV full-genome consensus sequences from the assemblies and ORF3 sequences obtained by Sanger sequencing have been submitted to GenBank Nucleotide Database (https://www.ncbi.nlm.nih.gov/nucleotide/), with accession numbers as listed in Supplementary Table S3. The new sequence data from Faroese Islands and previously published data from Ireland and Norway are also available in GenBank Nucleotide Database, with accession numbers as listed in Supplementary Table S3. Remaining sequence data from detection of defect viral genomes will be shared on reasonable request to the corresponding author. All data on histology scoring and from real-time PCR are available in the article and in its online supplementary material.
