# Supplementary material for: Unraveling the genomic landscape of piscine myocarditis virus: mutation frequencies, viral diversity and evolutionary dynamics in Atlantic salmon
Source: Virus Evol. 2024 Nov 21;10(1):veae097. doi: 10.1093/ve/veae097 (PMC11665822; doi:10.1093/ve/veae097)
Supplement: veae097_Supp [file veae097_supp.zip › veae097_Supp/suppl_data/Amono et al - Supplementary Fig S1.pdf]

Supplementary

Fig. S1 – Pairwise comparison of 34 complete or near complete PMCV full genomes from eight CMS cases and the reference genome from PMCV AL V-708 isolate

Percentages of identical nucleotides from pairwise comparisons of PMCV genome sequences are presented above the diagonal. Similarly, the number of divergent nucleotides (of 6688nt) from the pairwise comparisons are shown below the diagonal. Color shades from red to blue indicate highest to lowest pairwise sequence identities, respectively. Asterisks mark the two most diverging genome sequences.

		1	2	3	4	5	6	7	8	9	10	11	12	13	14	15	16	17	18	19	20	21	22	23	24	25	26	27	28	29	30	31	32	33	34	35
PMCV AL V708 reference isolate	1		99.66	99.66	99.66	99.60	99.66	99.63	99.51	99.60	99.51	99.61	99.51	99.57	99.54	99.69	99.72	99.49	99.58	99.70	99.78	99.72	99.51	99.52	99.54	99.52	99.58	99.46	99.54	99.54	99.54	99.64	99.61	99.52	99.52	99.52
A No2011-F17	2	23		99.94	99.94	99.52	99.64	99.67	99.43	99.64	99.43	99.66	99.43	99.46	99.58	99.64	99.66	99.48	99.63	99.66	99.70	99.61	99.40	99.48	99.58	99.57	99.63	99.52	99.58	99.58	99.58	99.69	99.66	99.57	99.57	99.57
A No2011-F18	3	23	4		99.94	99.52	99.64	99.67	99.43	99.64	99.43	99.66	99.43	99.46	99.58	99.64	99.67	99.48	99.63	99.66	99.70	99.61	99.40	99.48	99.58	99.57	99.63	99.52	99.58	99.58	99.58	99.69	99.66	99.57	99.57	99.57
A No2011-F19	4	23	4	4		99.52	99.64	99.67	99.43	99.64	99.43	99.66	99.43	99.46	99.58	99.64	99.66	99.48	99.63	99.66	99.70	99.61	99.40	99.48	99.58	99.57	99.63	99.52	99.58	99.58	99.58	99.69	99.66	99.57	99.57	99.57
A No2011-F20	5	27	32	32	32		99.49	99.49	99.46	99.46	99.46	99.48	99.25	99.28	99.40	99.49	99.51	99.33	99.45	99.51	99.52	99.48	99.43	99.30	99.40	99.39	99.45	99.33	99.40	99.40	99.40	99.51	99.48	99.39	99.39	99.39
B Tr2017-F1	6	23	24	24	24	34		99.66	99.43	99.63	99.43	99.66	99.46	99.49	99.73	99.64	99.64	99.51	99.63	99.84	99.67	99.61	99.46	99.46	99.52	99.51	99.64	99.70	99.55	99.52	99.55	99.63	99.66	99.51	99.52	99.51
B Tr2017-F2	7	25	22	22	22	34	23		99.40	99.91	99.40	99.66	99.40	99.45	99.58	99.63	99.61	99.67	99.63	99.67	99.67	99.63	99.40	99.45	99.55	99.54	99.63	99.51	99.76	99.55	99.76	99.66	99.66	99.54	99.54	99.54
B Tr2017-F3	8	33	38	38	38	36	38	40		99.39	99.97	99.39	99.16	99.19	99.34	99.40	99.40	99.24	99.36	99.46	99.43	99.37	99.64	99.21	99.31	99.30	99.39	99.27	99.31	99.31	99.31	99.42	99.42	99.30	99.30	99.30
B Tr2017-F4	9	27	24	24	24	36	25	6	41		99.37	99.63	99.37	99.42	99.55	99.58	99.61	99.63	99.60	99.64	99.67	99.60	99.37	99.42	99.52	99.51	99.60	99.49	99.76	99.52	99.78	99.63	99.63	99.52	99.51	99.51
B Tr2017-F19	10	33	38	38	38	36	38	40	2	42		99.39	99.16	99.22	99.34	99.40	99.40	99.24	99.36	99.46	99.43	99.37	99.64	99.21	99.31	99.30	99.39	99.27	99.31	99.31	99.31	99.42	99.42	99.30	99.30	99.30
B Tr2017-F20	11	26	23	23	23	35	23	23	41	25	41		99.39	99.42	99.60	99.60	99.60	99.49	99.64	99.67	99.66	99.57	99.39	99.46	99.54	99.52	99.64	99.52	99.60	99.54	99.60	99.64	99.67	99.52	99.52	99.52
C No2017-F5	12	33	38	38	38	50	36	40	56	42	56	41		99.85	99.37	99.37	99.42	99.21	99.36	99.45	99.61	99.37	* 99.15	99.75	99.34	99.33	99.36	99.27	99.31	99.34	99.33	99.45	99.39	99.33	99.33	99.33
C No2017-F9	13	29	36	36	36	48	34	37	54	39	52	39	10		99.40	99.43	99.46	99.27	99.39	99.49	99.64	99.43	99.19	99.75	99.37	99.36	99.39	99.30	99.36	99.37	99.36	99.48	99.42	99.36	99.36	99.36
D2 Tr2017-F2	14	31	28	28	28	40	18	28	44	30	44	27	42	40		99.52	99.52	99.39	99.57	99.72	99.61	99.51	99.34	99.42	99.46	99.45	99.57	99.60	99.49	99.46	99.49	99.57	99.60	99.45	99.45	99.45
D2 Tr2017-F11	15	21	24	24	24	34	24	25	40	28	40	27	42	38	32		99.91	99.48	99.57	99.66	99.67	99.88	99.40	99.42	99.70	99.69	99.57	99.45	99.52	99.70	99.52	99.75	99.60	99.69	99.69	99.69
D2 Tr2017-F12	16	19	23	22	23	33	24	26	40	26	40	27	39	36	32	6		99.48	99.58	99.66	99.70	99.91	99.40	99.45	99.66	99.64	99.57	99.51	99.54	99.66	99.54	99.72	99.60	99.64	99.64	99.64
D3 Tr2017-F13	17	34	35	35	35	45	33	22	51	25	51	34	53	49	41	35	35		99.43	99.52	99.51	99.48	99.27	99.25	99.36	99.34	99.43	99.31	99.55	99.36	99.55	99.46	99.46	99.34	99.34	99.34
D3 Tr2017-F14	18	28	25	25	25	37	25	25	43	27	43	24	43	41	29	29	28	38		99.64	99.64	99.54	99.36	99.40	99.54	99.52	99.76	99.54	99.60	99.54	99.60	99.64	99.79	99.52	99.52	99.52
D3 Tr2017-F15	19	20	23	23	23	33	11	22	36	24	36	22	37	34	19	23	23	32	24		99.69	99.66	99.49	99.49	99.54	99.52	99.66	99.66	99.57	99.54	99.57	99.64	99.67	99.52	99.54	99.52
E Ve2017-F2	20	15	20	20	20	32	22	22	38	22	38	23	26	24	26	22	20	33	24	21		99.67	99.43	99.64	99.58	99.57	99.64	99.55	99.60	99.58	99.60	99.69	99.66	99.57	99.58	99.57
E Ve2017-F4	21	19	26	26	26	35	26	25	42	27	42	29	42	38	33	8	6	35	31	23	22		99.37	99.42	99.64	99.63	99.54	99.42	99.55	99.64	99.55	99.70	99.57	99.63	99.63	99.63
F MR2017-F8	22	33	40	40	40	38	36	40	24	42	24	41	* 57	54	44	40	40	49	43	34	38	42		99.19	99.28	99.27	99.39	99.28	99.31	99.28	99.31	99.39	99.42	99.27	99.28	99.27
G TF2018_F1	23	32	35	35	35	47	36	37	53	39	53	36	17	17	39	39	37	50	40	34	24	39	54		99.36	99.34	99.40	99.31	99.36	99.36	99.37	99.46	99.43	99.34	99.34	99.34
H Tr2018-F1	24	31	28	28	28	40	32	30	46	32	46	31	44	42	36	20	23	43	31	31	28	24	48	43		99.99	99.54	99.39	99.46	100.00	99.46	99.90	99.57	99.99	99.99	99.99
H Tr2018-F2	25	32	29	29	29	41	33	31	47	33	47	32	45	43	37	21	24	44	32	32	29	25	49	44	1		99.52	99.37	99.45	99.99	99.45	99.88	99.55	99.97	99.97	99.97
H Tr2018-F3	26	28	25	25	25	37	24	25	41	27	41	24	43	41	29	29	29	38	16	23	24	31	41	40	31	32		99.52	99.57	99.54	99.57	99.64	99.97	99.52	99.52	99.52
H Tr2018-F4	27	36	32	32	32	45	20	33	49	34	49	32	49	47	27	37	33	46	31	23	30	39	48	46	41	42	32		99.43	99.39	99.46	99.49	99.55	99.37	99.39	99.37
H Tr2018-F5	28	31	28	28	28	40	30	16	46	16	46	27	46	43	34	32	31	30	27	29	27	30	46	43	36	37	29	38		99.46	100.00	99.57	99.60	99.46	99.45	99.45
H Tr2018-F6	29	31	28	28	28	40	32	30	46	32	46	31	44	42	36	20	23	43	31	31	28	24	48	43	0	1	31	41	36		99.46	99.90	99.57	99.99	99.99	99.99
H Tr2018-F7	30	31	28	28	28	40	30	16	46	15	46	27	45	43	34	32	31	30	27	29	27	30	46	42	36	37	29	36	0	36		99.57	99.60	99.46	99.46	99.45
H Tr2018-F8	31	24	21	21	21	33	25	23	39	25	39	24	37	35	29	17	19	36	24	24	21	20	41	36	7	8	24	34	29	7	29		99.67	99.88	99.88	99.88
H Tr2018-F9	32	26	23	23	23	35	23	23	39	25	39	22	41	39	27	27	27	36	14	22	23	29	39	38	29	30	2	30	27	29	27	22		99.55	99.55	99.55
H Tr2018-F10	33	32	29	29	29	41	33	31	47	32	47	32	45	43	37	21	24	44	32	32	29	25	49	44	1	2	32	42	36	1	36	8	30		99.97	99.97
H Tr2018-F11	34	32	29	29	29	41	32	31	47	33	47	32	45	43	37	21	24	44	32	31	28	25	48	44	1	2	32	41	37	1	36	8	30	2		99.97
H Tr2018-F12	35	32	29	29	29	41	33	31	47	33	47	32	45	43	37	21	24	44	32	32	29	25	49	44	1	2	32	42	37	1	37	8	30	2	2	
