# Supplementary material for: Unraveling the genomic landscape of piscine myocarditis virus: mutation frequencies, viral diversity and evolutionary dynamics in Atlantic salmon
Source: Virus Evol. 2024 Nov 21;10(1):veae097. doi: 10.1093/ve/veae097 (PMC11665822; doi:10.1093/ve/veae097)

Supplementary

Fig. S2 – Phylogenetic trees generated from ORF 1 and ORF2 sequences

A) Phylogenetic analysis of 98 complete or near complete PMCV ORF1 sequences from Norway and the Faroe Islands. The Norwegian sequences (Case A-H) are color-coded by case origin and with the year of sampling as indicated and follow the color-coding and unique IDs used in Figs. 1, 3, 4 and Supplementary Fig. S2B. Previously published Norwegian sequences from 2009-2010 and sequences from the Faroe Islands are given as indicated using a single color, independent of case origin. The Norwegian PMCV reference genome sequence AL V-708 from 2007 is highlighted in black. All bootstrap values 70 or above are shown in red color. The tree has been rooted against the wild salmon PMCV ORF1 sequence from the Faroe Islands for display purposes.

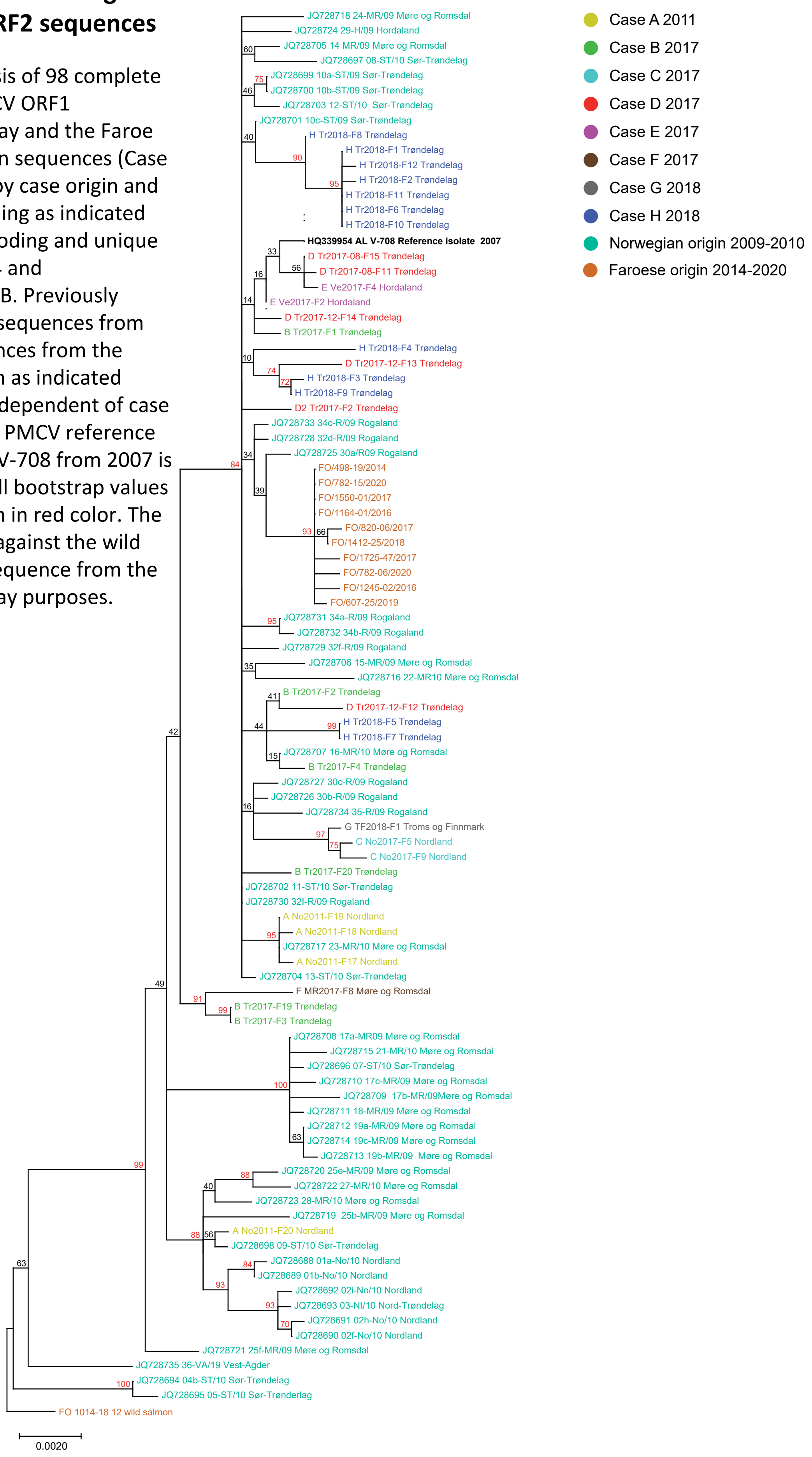

B) Phylogenetic analysis of 46 complete or near complete PMCV ORF2 sequences. The ORF2 sequences from Norwegian Cases A-H are color-coded as indicated and follow the color-coding and unique ID used in Fig. 1, 3, and 4, and Supplementary Fig. S2A. Sequences from the Faroe Islands are given as indicated using a single color, independent of case origin. The Norwegian PMCV reference genome sequence AL V-708 from 2007 is highlighted in black. All bootstrap values 70 or above are shown in red color. The tree has been rooted against the wild salmon PMCV ORF2 sequence from the Faroe Islands for display purposes

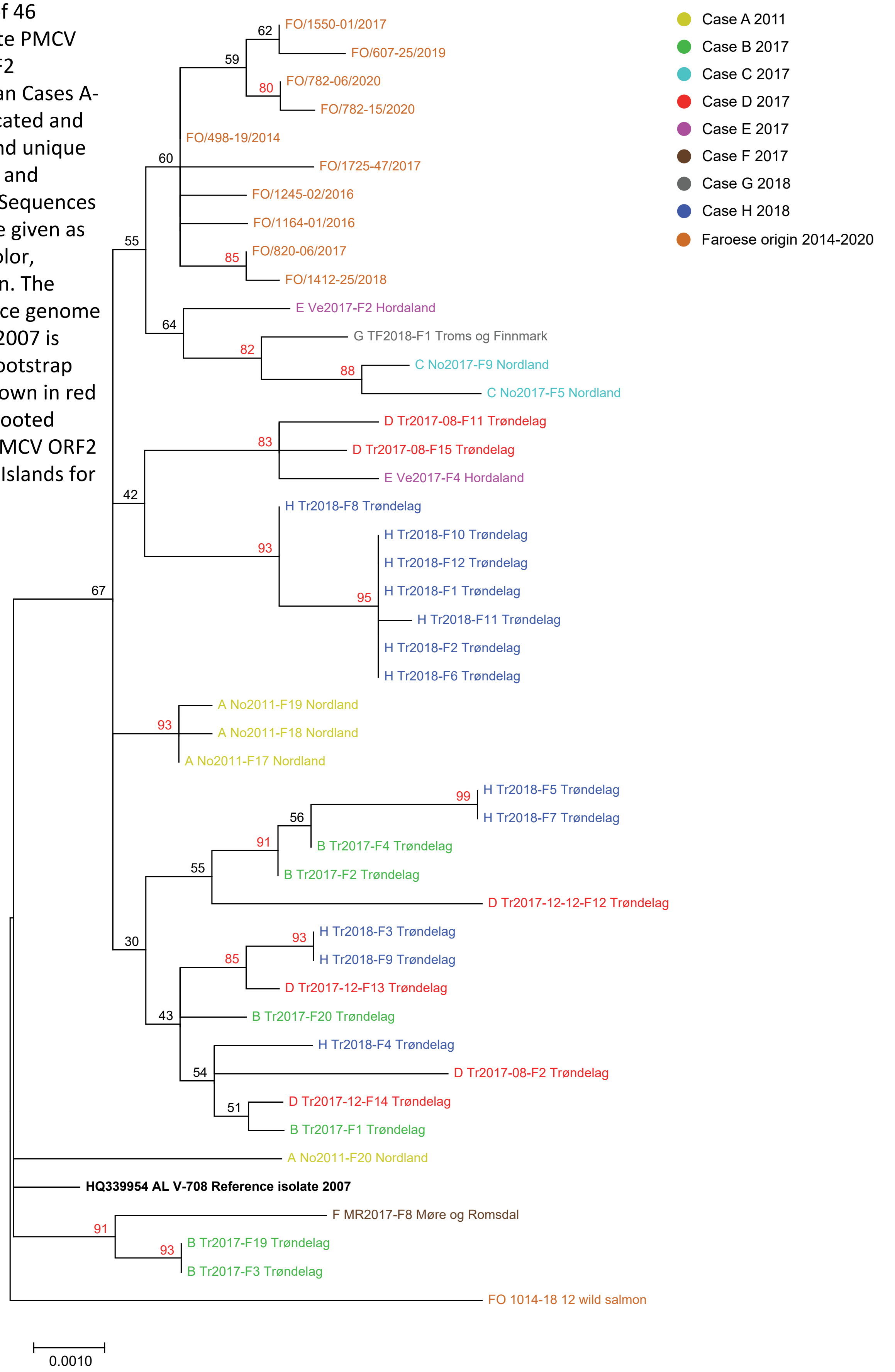

Supplement: veae097_Supp [file veae097_supp.zip › veae097_Supp/suppl_data/Amono et al - Supplementary Fig S2.pdf]
