# Supplementary material for: Unraveling the genomic landscape of piscine myocarditis virus: mutation frequencies, viral diversity and evolutionary dynamics in Atlantic salmon
Source: Virus Evol. 2024 Nov 21;10(1):veae097. doi: 10.1093/ve/veae097 (PMC11665822; doi:10.1093/ve/veae097)
Supplement: veae097_Supp [file veae097_supp.zip › veae097_Supp/suppl_data/Amono et al - Supplementary Fig S3.pdf]

Supplementary

Fig. S3 – Available PMCV genomic sequences from RNA seq on samples from Ex B

Alignment of available PMCV sequences from the field samples Case G F12, 16 and 20 pooled for the challenge material, the resulting challenge material, and from challenged fish from all time points. The genomic position is shown on top. PMCV AL V-708 isolate sequence is included for reference. Nucleotides deviating from the reference are indicated and are equal among all samples with available sequences, except for genomic positions 101 and 259 (5'UTR) and 5832 and 5884 (ORF3). A single dot represents no difference to reference. However, combined with a nucleotide, this indicates the reference nucleotide and a nucleotide differing from the reference were both found in significant amounts among the reads from RNAseq. Black nts – synonymous mutation, orange nts – non-synonymous mutation, grey background – sequencing was not performed (Case G samples), or the RNAseq data resulted in low or no coverage (challenge samples).

		5'UTR		ORF1			link[1-2]	ORF2						UTR[2-3]	ORF3				3'UTR
		101	259	1283	1751	2733	All	3173	3749	3869	4577	4681	5147	All	5575	5580	5832	5884	All
**AL V-708 (ref)**		C	C	C	C	A		T	T	A	T	G	T		C	A	A	G	
**Case G**	F12														T	T	G	.	
	F16														T	T	.	.	
	F20														T	T	.	A	
**Challenge material**			T	G	T	G	.	C	C	G	C	A	C	.	T	T	.	A	.
**6 wpc**	F1	.	T		T	G	.	C	C	G	C	A	C	.	T	T	.		
	F2	.	T		T	G	.	C	C	G	C	A	C	.	T	T	.	A	.
	F3	.	./T			G	.	C	C	G			C	.	T	T		A	.
	F4	.	.	G	T		.	C	C	G	C	A	C	.	T	T	.	.	.
	F5	.	T				.	C	C	G		A	C	.	T	T	.	./A	.
	F6	.	T		T	G	.	C	C	G	C	A	C	.	T	T	.	./A	.
	F7	.	T	G	T	G	.	C	C	G		A	C	.	T	T	.	A	.
	F8	.	T		T		.	C	C	G	C	A	C	.	T	T	.	A	.
	F9	.	.		T		.	C	C	G	C	A	C	.	T	T	.	A	.
	F10	.			T				C									.	.
**8 wpc**	F1	T		G	T	G	.	C	C	G	C	A	C	.	T	T	.	.	.
	F2	.	T	G	T	G	.	C	C	G	C	A	C	.	T	T	.	A	.
	F3	.	T		T	G	.	C	C	G	C	A	C	.	T	T	.	A	.
	F4	.	T	G	T	G	.	C	C	G	C	A	C	.	T	T	.	.	.
	F5	.	T		T	G	.	C	C	G	C	A	C	.	T	T	.	A	.
	F6	.	T	G	T	G	.	C	C	G	C	A	C	.	T	T	.	A	.
	F7	.	.	G	T	G	.	C	C	G	C	A	C	.	T	T	.	.	.
	F8	.	T		T	G	.	C	C	G	C	A	C	.	T	T	.	A	.
	F9	.	T	G	T	G	.	C	C	G	C	A	C	.	T	T	.	A	.
	F10	.	T	G	T	G	.	C	C	G	C	A	C	.	T	T	.	A	.
**10 wpc**	F1	.			T		.		C	G		A	C	.	T	T	.	A	.
	F2	.	T	G	T	G	.	C	C	G	C	A	C	.	T	T	.	A	.
	F3	.	T		T		.	C	C	G		A	C	.	T	T	.	A	.
	F4	.	T		T		.	C	C	G		A	C	.	T	T	.	A	.
	F5	.	T	G	T	G	.	C	C	G	C	A	C	.	T	T	.	A	.
	F6	.	./T	G	T	G	.	C	C	G	C	A	C	.	T	T	.	A	.
	F7	.	T	G	T		.		C	G	C		C	.	T	T	.		
	F8	.					.	C	C	G				.	T	T	.		
	F9	.	T		T		.	C	C	G	C	A	C	.	T	T	.	A	.
	F10	.	.		T	G	.	C	C	G	C	A	C	.	T	T	.	A	.
