# Supplementary material for: Unraveling the genomic landscape of piscine myocarditis virus: mutation frequencies, viral diversity and evolutionary dynamics in Atlantic salmon
Source: Virus Evol. 2024 Nov 21;10(1):veae097. doi: 10.1093/ve/veae097 (PMC11665822; doi:10.1093/ve/veae097)
Supplement: veae097_Supp [file veae097_supp.zip › veae097_Supp/suppl_data/Amono et al - Supplementary Material S1.pdf]

Supplementary Material S1

Analysis of a putative association between specific PMCV variants, degree of heart lesion scores and PMCV RNA levels

Heart tissue sections available from a selection of the field cases in the present study, were evaluated for characteristic CMS lesions in the atrium and compact and spongy layers of the ventricle, and the degree of lesions scored from 0 (no lesions observed) to 4 (severe lesions). The scores are summarized in the table below with Ct levels representing PMCV RNA levels, measured by real-time PCR on parallel tissue samples. The PMCV variants found in each sample are sorted into subgroups per case representing main variants, based on sequence similarities apparent from multiple sequence alignments and phylogenetic analyses as described in manuscript.

Table - PMCV RNA levels and degree of heart tissue lesions in fish from different CMS cases grouped by PMCV variant similarity

Case	Main variant group[a]	Sample name	PMCV RNA levels [Ct]	Histology score[b] Atrium	Histology score[b] Ventricle
A	A I	A No2011-F17	17.61	4	4
		A No2011-F18	16.28	4	4
		A No2011-F19	15.69	4	3
	A II	A No2011-F20	19.78	4	4
B	B I	B Tr2017-F2	19.11	3	4
		B Tr2017-F4	15.74	4	4
	B II	B Tr2017-F1	19.54	0	0
	B III	B Tr2017-F20	17.97	4	4
	B IV	B Tr2017-F3	16.93	4	4
		B Tr2017-F19	20.34	4	4
D	D I	D2 Tr2017-F11	26.35	3	2
		D2 Tr2017-F15	17.92	1	0
	D II	D3 Tr2017-F12	23.31	2	2
	D III	D2 Tr2017-F2	23.33	4	3
		D3 Tr2017-F14	26.34	2	2
	D IV	D3 Tr2017-F13	20.18	4	4
E	E I	E Ve2017-F2	21.38	4	3
	E II	E Ve2017-F4	21.84	2	2
F	F I	F MR2017-F8	20.47	1	1
H	H I	H Tr2018-F1	19.64	4	4
		H Tr2018-F2	18.65	3	4
		H Tr2018-F11	19.65	n.a.	4
	H I+II mix	H Tr2018-F6	36.27	4	4
		H Tr2018-F12	17.46	4	4
		H Tr2018-F10	19.08	n.a.	4
		H Tr2018-F8	16.00	3	4
	H II	H Tr2018-F9	17.24	4	4
		H Tr2018-F3	n.a.	4	4
	H III	H Tr2018-F5	17.34	4	4
		H Tr2018-F7	18.07	4	4
	H IV	H Tr2018-F4	20.74	4	4

[a] Samples from each case are sorted into groups based on sequence similarities apparent from multiple sequence alignments and phylogenetic analyses (Fig. 3, for Case H also Fig. 5), and groups are named according to case and numbering.
[b] Heart tissue lesions described by scoring of atrium and ventricle compartments from 0 (no lesions) to 4 (severe lesions)

The sum of atrium/ventricle scores in these field samples is plotted against obtained Ct values with a resulting slope in the trendline 0.41 (41% of variability in heart scores explained by variation in Ct values), see figure below. A regression analysis shows that there is an association between scores and Ct values, as also shown previously (Haugland, et al. 2011; Wiik-Nielsen, et al. 2016; Fritsvold, et al. 2021; Fritsvold, et al. 2022).

Figure – PMCV RNA levels (Ct values) plotted against degree of heart tissue lesions (sum of atrium/ventricle scores) in fish from different CMS cases

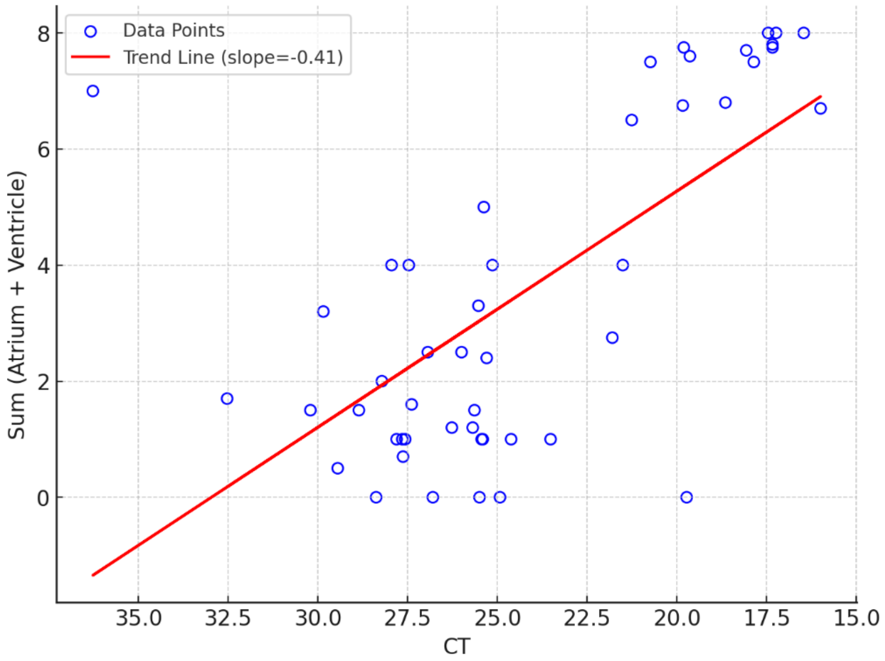

To identify PMCV variants with potential differences in virulence that could be found in specific cases or related to defined clusters seen from phylogeny, we individually evaluated a possible association between the heart lesion score and levels of PMCV RNA to specific PMCV variants. We found no evidence to support whether specific virus variants could be characterized as high or low virulent. Cases A, B, and H include fish individuals characterized by severe heart lesions (mean score 4) and high levels of virus RNA, which could suggest variants of higher virulence. Still, we cannot conclude whether specific variants are more virulent than others, as heart lesion scores, PMCV RNA levels, and full genome sequence information were most studied at late, but random time points after the initial virus entry into the population. A complete evaluation of virulence caused by genetic factors of the virus necessitates *in vitro* and/or *in vivo* time course studies under controlled experimental conditions.

References:

- Fritsvold C, Mikalsen AB, Haugland Ø, Tartor H, Sindre H. 2022. Characterization of early phases of cardiomyopathy syndrome pathogenesis in Atlantic salmon (*Salmo salar* L.) through various diagnostic methods. J Fish Dis.
- Fritsvold C, Mikalsen AB, Poppe TT, Taksdal T, Sindre H. 2021. Characterization of an outbreak of cardiomyopathy syndrome (CMS) in young Atlantic salmon, *Salmo salar* L. J Fish Dis 44:2067-2082.
- Haugland Ø, Mikalsen AB, Nilsen P, Lindmo K, Thu BJ, Eliassen TM, Roos N, Rode M, Evensen Ø. 2011. Cardiomyopathy syndrome of Atlantic salmon (*Salmo salar* L.) is caused by a dsRNA virus of the *Totiviridae* family. J Virol 85:5275-5286.
- Wiik-Nielsen J, Alarcon M, Jensen BB, Haugland O, Mikalsen AB. 2016. Viral co-infections in farmed Atlantic salmon, *Salmo salar* L., displaying myocarditis. J Fish Dis 39:1495-1507.
