# Supplementary material for: Unraveling the genomic landscape of piscine myocarditis virus: mutation frequencies, viral diversity and evolutionary dynamics in Atlantic salmon
Source: Virus Evol. 2024 Nov 21;10(1):veae097. doi: 10.1093/ve/veae097 (PMC11665822; doi:10.1093/ve/veae097)
Supplement: veae097_Supp [file veae097_supp.zip › veae097_Supp/suppl_data/Amono et al - Supplementary Table S1.pdf]

Supplementary

Table S1 – Overview of the experimental handling of individual samples from all cases

Case	Method for nucleic acid extraction[a]	Real-time PCR method used
**<u>Field outbreak – production sites</u>**		
**A**	RNA by Manual 1 (PCR+Sanger) + Manual 2 (NGS)	Two-step[b]
**B**	RNA by Manual 1 (PCR+Sanger) + Manual 2 (NGS)	Two-step[b]
**C**	RNA by Robot (PCR + Sanger) + Manual 2 (NGS)	One-step[c]
**D1**	RNA by Robot (PCR + Sanger)	One-step[c]
**D2**	RNA by Robot (PCR+Sanger) + Manual 2 (NGS)	One-step[c]
**D3**	RNA by Robot (PCR + Sanger) + Manual 2 (NGS)	One-step[c]
**E**	RNA by Manual 1 (PCR+Sanger) + Manual 2 (NGS)	Two step[b]
**F**	RNA by Robot (PCR + Sanger) + Manual 2 (NGS)	One-step[c]
**G**	RNA by Robot (PCR + Sanger) + Manual 2 (NGS)	One-step[c]
**H**	RNA by Manual 1 (PCR+Sanger) + Manual 2 (NGS)	Two-step[b]
**I**	Nucleic acids, PatoGen AS (PCR+Sanger)	PatoGen[d]
**J**	Nucleic acids, PatoGen AS (PCR+Sanger)	PatoGen[d]
**K**	Nucleic acids, PatoGen AS (PCR+Sanger)	PatoGen[d]
**L**	Nucleic acids, PatoGen AS (PCR+Sanger)	PatoGen[d]
**<u>Field outbreaks – brood fish sites</u>**		
**Bf A**	Nucleic acids, Pharmaq Analytiq AS (PCR+Sanger)	One-step[c]
**<u>Challenge trials</u>**		
**Ex A**	RNA by Manual 1 (PCR+Sanger)	Two-step[b]
**Ex B**	RNA by Manual 1 (PCR+Sanger) + Manual 2 (NGS)	Two-step[b]

[a] "Manual 1" represents manual extraction using the RNeasy® Fibrous Tissue Mini Kit (Qiagen, Aarhus, Denmark). "Manual 2" the QIAamp Viral RNA Kit (Qiagen, Aarhus, Denmark). "Robot" represents automized procedure including Reliaprep simplyRNA HT system (Promega) on Biomek 4000 (Beckman Coulter). Total nucleic acid extraction was performed using commercial services offered by PatoGen AS, Ålesund, Norway or Pharmaq Analytiq AS, Bergen, Norway, as indicated.

SuperScript™ III Platinum®Two-Step qRT-PCR Kit with SYBR® Green (Invitrogen) with primers and setup as described by Haugland *et al.* 2011.

[b] QuantiFast SYBR Green RT-PCR Kit (Qiagen) with primers CMS-qPCR-F4 and CMS-qPCR-R4 (Table S1).

[c] PCR analyses performed by PatoGen AS (Norway). The real-time PCR assay targeting PMCV is validated to ISO17025 standards by PatoGen AS.
