# Supplementary material for: Unraveling the genomic landscape of piscine myocarditis virus: mutation frequencies, viral diversity and evolutionary dynamics in Atlantic salmon
Source: Virus Evol. 2024 Nov 21;10(1):veae097. doi: 10.1093/ve/veae097 (PMC11665822; doi:10.1093/ve/veae097)
Supplement: veae097_Supp [file veae097_supp.zip › veae097_Supp/suppl_data/Amono et al - Supplementary Table S2.pdf]

# Supplementary

## Table S2 – Primers and PCR amplicons

Primer combinations used with primer sequence, resulting amplicon size and genome positions, and a short description of the genomic content of amplicon.

| | | | | Amplicon | | |
|---|---|---|---|---|---|---|
| **Forward primer** | | **Reverse primer** | | **Size** [bp] | **Genome positions** [nts] | **Main genomic target** |
| **Real-time PCR** | | | | | | |
| **CMS-qPCR-F4** | AGACTACTCGCGGTTCGATT | **CMS-qPCR-R4** | CCGTCAATGTACACCGTTTG | 144 | 3959-4102 | Small part of ORF2 |
| **PMCV-F[a]** | ACACCAGGTGACCGAAAAG | **PMCV-R[a]** | TCCAGTGCCTTGATGTCTG | 237 | 3473-3709 | Small part of ORF2 |
| **Amplification for cloning and Sanger sequencing of insert in plasmid clones** | | | | | | |
| **ORF3-NheI-F[b]** | GGCAGCTAGCCGGATGTCAAACAAGATGAA | **ORF3-XhoI-R[b]** | CCGGCTCGAGATCTAGACTCCTACTCTGAA | 934 | 5529-6462 | Full ORF3 |
| **Amplification for Sanger sequencing of PCR amplicons** | | | | | | |
| **PMCV-ORF3-F1** | GGGACTTCACTTACCGCAGA | **PMCV-ORF3-R1** | TCGTTCTTGGCTTCTTGTGA | 685 | 5438-6122 | ORF3 5'end |
| **PMCV-ORF3-F2** | TAGGAACGGGTTCCATCTTG | **PMCV-ORF3-R2** | CGACCTATTTGTCGGTCTGG | 705 | 5856-6560 | ORF3 3'end |
| **PMCV-HMR-F1** | GGATTGTTCCAATGTTCAGGC | **PMCV-HMR-R1** | CCGAATGAAGCAAGATGGAAC | 286 | 5600-5885 | ORF3 part of 5'end |
| **PMCV-F[a]** | ACACCAGGTGACCGAAAAG | **PMCV ORF3-R2** | CGACCTATTTGTCGGTCTGG | 3088 | 3473-6560 | ORF2-UTR$^{2-3}$-ORF3 |
| **PMCV-ORF1-F1** | GTAGAAAGGTGGGTCGAGC | **PMCV-ORF1-R2** | GTCTCTCTCGTTGGCTCAG | 2968 | 319-3286 | ORF1 |
| **PMCV-ORF2-F1.1** | GCCAACCAACAACAGACAC | **PMCV-ORF2-R2** | GCCTCTGCGGTAAGTGAAG | 2486 | 2975-5460 | ORF2 |
| **PMCV-ORF2-F2.1** | GCGGATGGAGATGGACAAG | **PMCV-ORF3-R2** | CGACCTATTTGTCGGTCTGG | 2423 | 4138-6560 | 3'ORF2-ORF3 |

[a]Haugland *et al.* 2011.

[b]Originally designed to add 5' NheI and 3' XhoI restriction enzyme sites. The 5'sequence overhangs including the restriction enzyme sites not specific to PMCV, are underlined.
