# Supplementary material for: Unraveling the genomic landscape of piscine myocarditis virus: mutation frequencies, viral diversity and evolutionary dynamics in Atlantic salmon
Source: Virus Evol. 2024 Nov 21;10(1):veae097. doi: 10.1093/ve/veae097 (PMC11665822; doi:10.1093/ve/veae097)
Supplement: veae097_Supp [file veae097_supp.zip › veae097_Supp/suppl_data/Amono et al - Supplementary Table S4.pdf]

Supplementary

Table S4 - Diversity and selection measures for the PMCV genome, individual ORFs, and UTRs within Case H

Total number of variable positions found relative to the PMCV reference isolate AL V-708, percentage of variable positions to total positions, number of sequence variants found, sequence diversity, nucleotide diversity (π), and Tajima's D values for the PMCV genome, individual ORFs, and UTRs. dN/dS values are calculated for the individual ORFs. The values were obtained from the 12 genomes generated from Case H in the present study and describe the measures for a single case as a comparison to Table 2 in manuscript based on all 34 genomes from eight cases. Sequence diversity describes the uniqueness of a particular sequence variant in a given population, i.e., the probability that two sequences randomly sampled are different (varies between 0 (all sequences are equal) and 1 (all are different)). π describes the average number of pairwise nucleotide differences between two randomly chosen sequences per position. Tajima's D is a measure that distinguishes between sequences evolving neutrally (Tajima's D = 0) or under a non-neutral process (Tajimas D ± 0). The ratio of non-synonymous substitutions per non-synonymous (dN) to the number of synonymous substitutions per synonymous (dS), dN/dS, describes the strength and mode of natural selection acting on coding regions (dN/dS =1 indicates neutral selection).

	**Complete genome**	**5'UTR**	**ORF1/ Capsid**	**link[1-2]**	**ORF2/ RdRp**	**UTR[2-3]**	**ORF3/ p33**	**3'UTR**
Genomic position	1-6688	1-444	445-3030	3031-3113	3114-5294	5295-5541	5542-6450	6451-6688
Total number of nts	6688	444	2586	83	2181	247	909	238
Number of variable nts	72	0	30	2	26	3	11	0
Variable nts of total (%)	1.1	0	1.2	2.4	1.2	1.2	1.2	0
Sequence variants[a]	9	1	8	3	5	4	6	1
Sequence diversity[a]	0.939	0	0.894	0.439	0.742	0.561	0.758	0
- SD of Seq. div.	0.058	0	0.078	0.158	0.116	0.154	0.122	0
Nucleotide diversity π[a]	0.0034	0	0.0037	0.0057	0.0042	0.0026	0.0031	0
- SD of Nucl. div.	0.0006	0.0000	0.0006	0.0022	0.0008	0.0009	0.0009	0.0006
Tajima's D value[ab]	-0.039	n.a.	0.083	-0.850	0.555	-1.180	-0.907	n.a.
dN/dS[a]	n.a.	n.a.	0.0391	n.a.	0.0427	n.a.	0.121	n.a.

[a]Analyses performed using the DnaSP v6 software. The number of sequence variants and variant diversity were provided from the software's resulting values of haplotypes (h) and haplotype (gene) diversity (Hd), respectively.
[b]None of these values were given by the software as significant.
SD – standard deviation. n.a. – not applicable.
